# Innovative biosynthesis, artificial intelligence-based optimization, and characterization of chitosan nanoparticles by *Streptomyces microflavus* and their inhibitory potential against *Pectobacterium carotovorum*

**DOI:** 10.1038/s41598-022-25726-w

**Published:** 2022-12-17

**Authors:** Noura El-Ahmady El-Naggar, Shimaa I. Bashir, Nashwa H. Rabei, WesamEldin I. A. Saber

**Affiliations:** 1 Department of Bioprocess Development, Genetic Engineering and Biotechnology Research Institute, City of Scientific Research and Technological Applications (SRTA-City), New Borg El-Arab City, Alexandria 21934 Egypt; 2 Department of Plant Protection and Biomolecular Diagnosis, Arid Land Cultivation Research Institute, City of Scientific Research and Technological Applications (SRTA-City), New Borg El-Arab City, Alexandria 21934 Egypt; 3 Microbial Activity Unit, Department of Microbiology, Soils, Water and Environment Research Institute, Agricultural Research Center, Giza, 12619 Egypt

**Keywords:** Nanoparticles, Applied microbiology

## Abstract

Microbial-based strategy in nanotechnology offers economic, eco-friendly, and biosafety advantages over traditional chemical and physical protocols. The current study describes a novel biosynthesis protocol for chitosan nanoparticles (CNPs), employing a pioneer *Streptomyces* sp. strain NEAE-83, which exhibited a significant potential for CNPs biosynthesis. It was identified as *Streptomyces microflavus* strain NEAE-83 based on morphological, and physiological properties as well as the 16S rRNA sequence (GenBank accession number: MG384964). CNPs were characterized by SEM, TEM, EDXS, zeta potential, FTIR, XRD, TGA, and DSC. CNPs biosynthesis was maximized using a mathematical model, face-centered central composite design (CCFCD). The highest yield of CNPs (9.41 mg/mL) was obtained in run no. 27, using an initial pH of 5.5, 1% chitosan, 40 °C, and a 12 h incubation period. Innovatively, the artificial neural network (ANN), was used for validating and predicting CNPs biosynthesis based on the trials data of CCFCD. Despite the high precision degree of both models, ANN was supreme in the prediction of CNPs biosynthesis compared to CCFCD. ANN had a higher prediction efficacy and, lower error values (RMSE, MDA, and SSE). CNPs biosynthesized by *Streptomyces microflavus* strain NEAE-83 showed in-vitro antibacterial activity against *Pectobacterium carotovorum*, which causes the potato soft rot. These results suggested its potential application for controlling the destructive potato soft rot diseases. This is the first report on the biosynthesis of CNPs using a newly isolated; *Streptomyces microflavus* strain NEAE-83 as an eco-friendly approach and optimization of the biosynthesis process by artificial intelligence.

## Introduction

Actinomycetes comprise a broad unique group of Gram-positive, and aerobic actinobacteria, having high GC content in the genome (69–73%). Actinomycetes produce branching substrate and aerial mycelium that develops into chains of spores by forming cross-walls in the multinucleate aerial filaments. This group of bacteria is distributed broadly in soil and has plentiful pigmentation patterns^1,2^. Owing to their capacity to produce numerous value-added secondary metabolites and several applications in biological processes, *Streptomyces* species are the most industrially important among actinomycetes^3–5^. One newly emerging and promising application of actinomycetes is their application in the biosynthesis of nanoparticles^1,6^.

In recent years, nanoparticles have attracted significant attention due to their fascinating properties^7^. In comparison to bulk materials, nanoparticles have a high reaction activity due to their greater surface area-to-volume ratio^8^. Generally, nanoparticles could be generated by chemical, physical, mechanical, or biological routes^9^.

There are non-biological methods that provide biocompatible nanostructured systems without the use of harmful or high-cost materials, such as chitosan or albumin with sodium tripolyphosphate (TPP)^10–12^. Despite this, a number of obstacles contribute to the limitations of the non-biological based methods including the high cost, the utilization of high pressure, energy, temperature, hazardous compounds, and big particle size^13^. The hazardous chemicals limit the use of nanoparticles in medical and clinical fields. Consequently, there is an urgent need to establish eco-friendly alternative methods to synthesize nanoparticles^9^. Besides, ecofriendly, and economic, microbial-based synthesis of nanoparticles offers cleanness, safeness, and fastness, furthermore, the reductive capabilities of the microbial metabolites can easily generate monodispersed nanoparticles^9^.

Chitosan is a deacetylated derivative of chitin, composed of a linear polysaccharide of linked (β1 → 4) residues of *N*-acetyl-2 amino-2-deoxy-d-glucose (glucosamine), and 2-amino-2-deoxy-d-glucose (*N*-acetyl-glucosamine). It is biodegradable, soluble in an aqueous acidic medium via primary amine protonation, and the free amino groups generate a positive charge on its polymeric chains^14,15^.

The renovation and usage of chitosan have attracted a growing interest in a diverse field e.g., the broad-spectrum antimicrobial properties were described against numerous pathogens^16^. Interestingly, chitosan was previously used for the synthesis of metallic nanoparticles as a reducing and/or stabilizing agent. What is more, chitosan itself has long been used as a material to produce chitosan nanoparticles. The natural preparation of chitosan nanoparticles (CNPs) has several favorable attributes e.g., biocompatibility, nontoxicity, biodegradability, and environmental safety, what is more, CNPs has a high permeability through biological membranes and therefore, preferred for a wide range of biological applications^14,15^.

The response surface methodology (RSM) is a modeling approach that provides an efficient statistical technique applied to optimize the variables and the process performance. RSM is cost-effective, reduces the overall number of experiments conducted to assess numerous variables, applicable, identifies the optimal conditions, and maintains a high level of precision for the highest yield as compared to the conventional method^17–19^.

Artificial neural network (ANN) is the central piece of artificial intelligence and one of the main teaching tools applied in machine learning^20,21^. Like the human brain, ANN is an advanced tool that can analyze and process data, thus efficiently building computational models with inter-communicated points (nodes) inside the hidden layer(s). This kind of modeling enables learning the data patterns and therefore, making accurate choices based on a given investigational data. Through ANN modeling, the network architecture is selected, then constructing the hidden layer(s) with enough neurons. Such a network starts learning and training processes until understands the data pattern. Finally, validation and verification of the resulting ANN model take place before approving the predictive model^20,21^.

The learning mechanism of ANN is based on diagnosing the diverse designs in the data to detect any differences and decide which pattern achieves the target, this process is controlled through intelligent backpropagation that generates the desired outturn model that attains the goal. The deep learning procedure is supposedly more truthful and can effectively substitute the other modeling policies^20,21^.

Blackleg and bacterial soft rot caused by *Pectobacterium carotovorum* (perversely called *Erwinia carotovora*) are among the most important diseases that cause an economic loss of potatoes and other vegetable crops through reduced yield and quality. Soft rot disease may affect potato tubers in the ground and under storage, as well^22,23^. *Pectobacterium carotovorum* is a gram-negative, aggressive necrotrophic bacteria that produce various plant cell wall-degrading enzymes, including pectinases, polygalacturonase, cellulases and protease, which results in the maceration of plant tissue and soft rot symptoms^24^. Tuber contamination is the main way for the disease to spread due to the ability of bacterial pathogens to colonize the tuber surface and remain symptomless^25^. The traditional strategy of soft rot management was restricted to the use of bactericides and antibiotics. Many of these compounds were banned in developed countries because of their hazards to ecosystems and public health^26^. Therefore, there is increasing interest in the development of new methods, such as using nanomaterials to limit the use of pesticides.

No evidence is available on the microbial conversion of chitosan into nanoparticles. To cover such chasm, a unique microbial system (*Streptomyces microflavus*) was developed together with ANN, as a novel modeling approach, to optimize the operation parameters of CNPs biosynthesis. The characterization of the biofabricated nanoparticles was documented.

## Results and discussion

Recent nanotechnology has occupied a unique situation in modern life, owing to the preferred features, especially when applied to biological systems. When compared to bulk materials, nanoparticles (NPs) display novel behaviors due to their small size, diverse shapes, as well as varied optical, thermal, electrical, and mechanical characteristics. Organic nanoparticles include those that are fabricated from organic components such as proteins, carbohydrates, lipids, polymers such as chitosan, and any other organic substance. Typically, these NPs are biodegradable and non-toxic^27^. The prospective field of application for organic NPs is determined by different criteria, including composition, surface shape, stability, carrying capacity, and others^27^.

Several protocols were stated for the manufacture of nanoparticles. Physical and chemical approaches, as well as mixture procedures, use highly concentrated stabilizers and reductants that are detrimental to both the environment and human health**.** The biological-based procedure has emerged with several advantages. The technique is a single-step process and so, environmentally friendly, non-toxic, and requires lower energy, further, the biological-based product has greater stability^28^. Nanoparticles synthesis based on microbial factories came superior to other methods. Most bio-fabrication of nanoparticles is limited to metal ions, and no describes of CNPs bio-fabrication were established on microbial bases. However, microbial factory techniques follow the green chemistry principles and the biosynthesized chitosan nanoparticles are stable, non-toxic, and free of potentially harmful chemical compounds^29,30^.

A total of ten morphologically dissimilar actinomycete strains were screened for their potential for chitosan nanoparticles biosynthesis. Among these isolates, 5 isolates exhibited an obvious ability to biosynthesize CNPs. Of them, the most promising isolate *Streptomyces* sp. strain NEAE-83 was selected for further studies. No previous effort, according to the best of the authors' knowledge, has described the synthesis of CNPs by *Streptomyces* sp. strain NEAE-83. A pioneer biofabrication process of CNPs was reported in the current study using a newly isolated *Streptomyces* sp. strain NEAE-83.

### UV/visible spectra of CNPs generated by *Streptomyces* sp. strain NEAE-83

**Figure 1:**
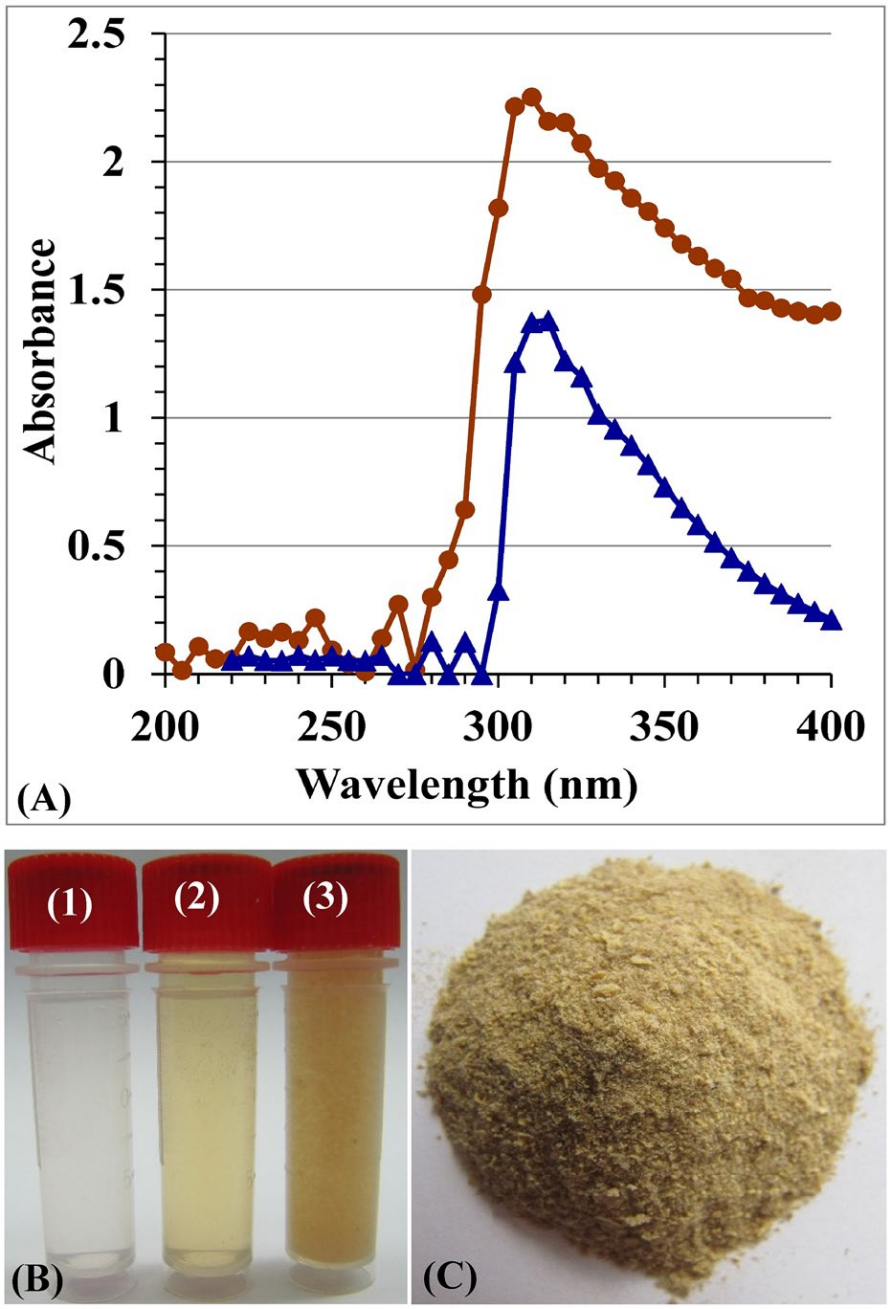
(**A**) UV/visible spectra of chitosan (blue line) and chitosan nanoparticles (red line), (**B**) Vials of chitosan solution (**B1**), *Streptomyces microflavus* strain NEAE-83 filtrate (**B2**), and the biosynthesized chitosan nanoparticles (**B3**), and (**C**) desiccated chitosan nanoparticles bio-synthesized using *Streptomyces microflavus* strain NEAE-83.

The bacterial fabrication pattern of CNPs was checked and examined in the UV/VIS spectra from 200 to 400 nm (Fig. 1A). The optical features of the generated CNPs biopolymer exhibited a solitary severe peak with the greatest absorbance at 310 nm. Figure 1B,C show three vials of chitosan: *Streptomyces* sp. strain NEAE-83 supernatant, and the CNPs, as well as the nanoparticles after extraction and drying.

The single peak absorbance at 310 nm CNPs by *Streptomyces* sp. strain NEAE-83 in the UV region lies within the previously reported range of 285 nm^30^ and 320 nm^15^. The UV/visible spectrum of CNPs is considered an indicator of the success of the biosynthesis process, where it is sharp in CNPs, but has a broader absorption band in chitosan^15,30^.

### Identification of *Streptomyces* sp. strain NEAE-83

**Figure 2:**
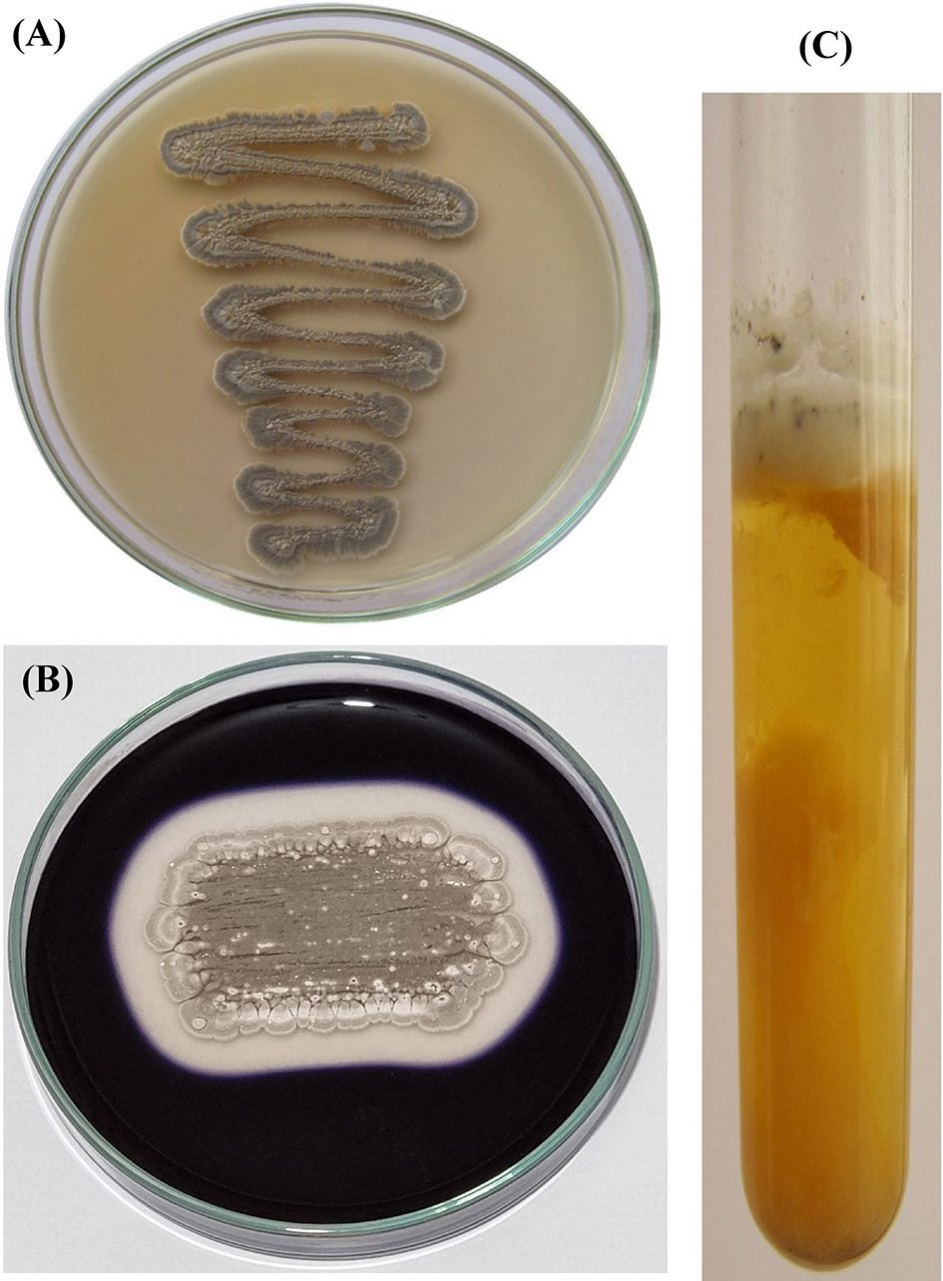
(**A**) Colored mycelium of *Streptomyces* sp. strain NEAE-83. grown on yeast extract-malt extract agar medium. (**B**) Clearance zone of hydrolyzed starch by extracellular *Streptomyces* sp. amylases of. (**C**) Milk coagulation and peptonization.

The current *Streptomyces* sp. strain NEAE**-**83 has been completely classified depending on its culture, morphology, cell physiology characteristics, and the 16S rRNA sequence. It was non-motile, aerobic, and formed long well-developed substrate mycelium. The reverse side of the colony was brown and the color of the mycelium was gray without diffusible pigments (Fig. 2A). The colonial morphology was consistent with the genus *Streptomyces*^31,32^.

**Figure 3:**
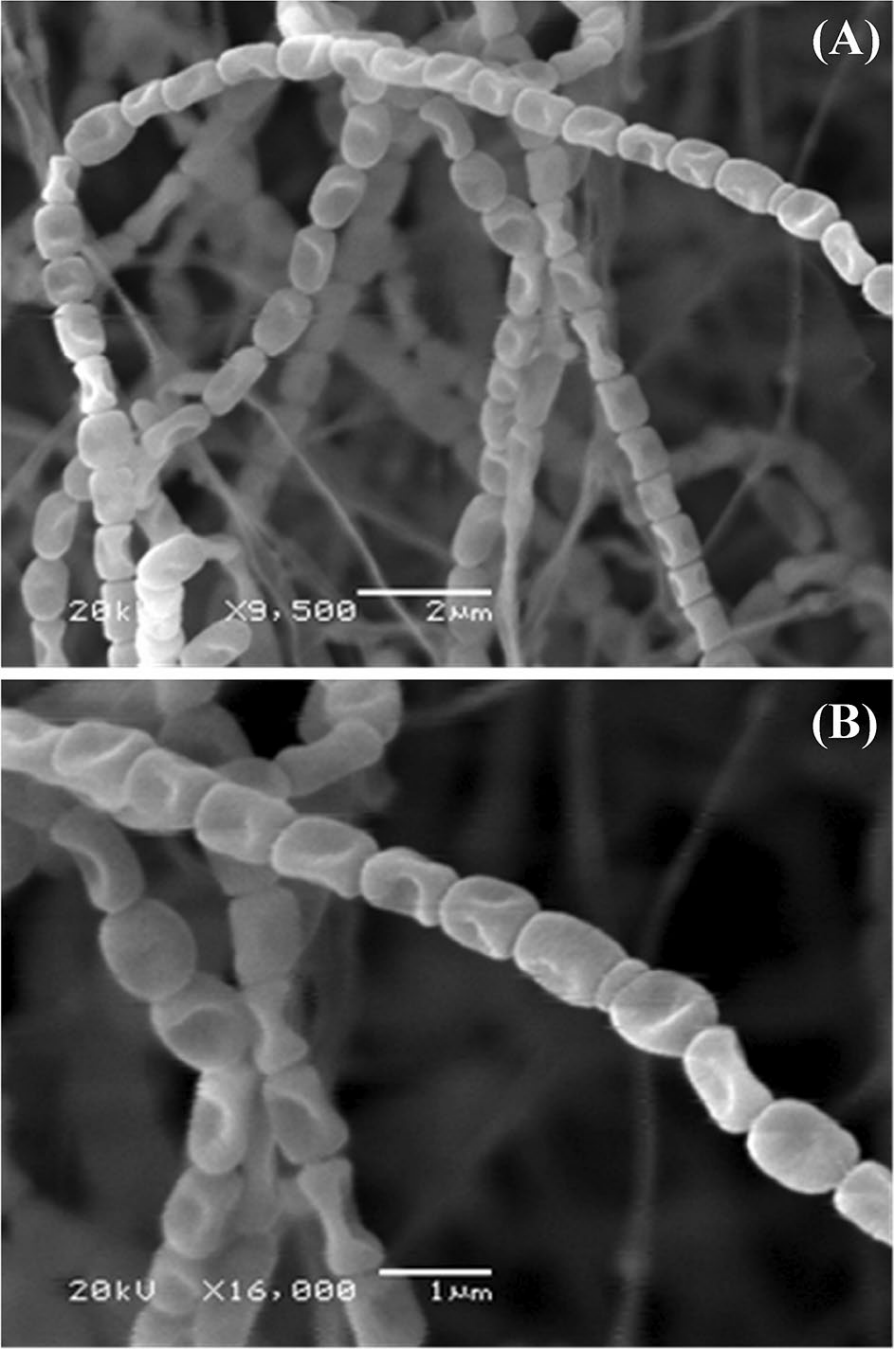
The ornamentation of the spore-chain morphology and spore-surface of *Streptomyces* sp. strain NEAE-83 at the magnification of ×9500 (**A**) and ×16,000 (**B**), as detected by scanning electron micrograph.

Scanning electron micrograph (Fig. 3) shows that aerial vegetative mycelia are abundant and well-developed and the aerial hyphae are long. It could be noticed that the aerial hyphae distinguished into Rectiflexibiles spore chains bearing more than 30 spores with smooth-surfaced, measuring 0.33 ± 0.04 × 0.89 ± 0.10 µm. The aerial spores are elongated.

### Physiological features

The data in Table 1 displayed the physiological features of *Streptomyces* sp. strain NEAE**-**83. Milk coagulation, peptonization, hydrolysis of starch, liquefaction of gelatin, protease, uricase, cellulase and chitosanase were positive, but L-asparaginase, nitrate reduction, melanoid pigment, and H_2_S production were negative. Optimal microbial development was at 30–37 °C and pH 7 with NaCl tolerance up to 3% (w/v). Diffusible pigments were not detected. These identification criteria are mostly following the genus *Streptomyces*^2,31,32^. Another, the *Streptomyces* sp. strain NEAE-83 exhibited no antimicrobial activity against all tested Gram-positive and Gram-negative bacteria and fungal strains.

**Table 1:** Morphological, physiological, and antimicrobial properties of *Streptomyces* NEAE-83.

Test	Reaction
**Morphology**	
Spore chain morphology	Rectiflexibiles
Spore shape	Elongated
Spore surface	Smooth
The reverse side of the colony	Brown
Color of spores	Gray
**Physiology**	
Diffusible pigment	None
Coagulation of milk	+
Peptonization of milk	+
Starch hydrolysis	+
Chitosanase	+
L-asparaginase	–
Protease	+
Uricase	+
Cellulase	+
Melanoid pigment	–
Nitrate reduction	–
Liquefaction of gelatin	+
Production of H_2_S	–
Tolerance of NaCl	Up to 3%
**Antimicrobial activity**	
Against Gram-positive and Gram-negative bacteria	–
Against fungal strains	–

Positive reaction; +, Negative reaction; –, Bacterial strains were *Staphylococcus aureus*, *Pseudomonas aeruginosa*, *Klebsiella*, *Escherichia coli*, Fungal strains were *Fusarium solani*, *Alternaria solani*, *Fusarium oxysporum*, *Bipolaris oryzae*, *Rhizoctonia solani.*

### Gene sequencing and phylogenetic analysis

**Figure 4:**
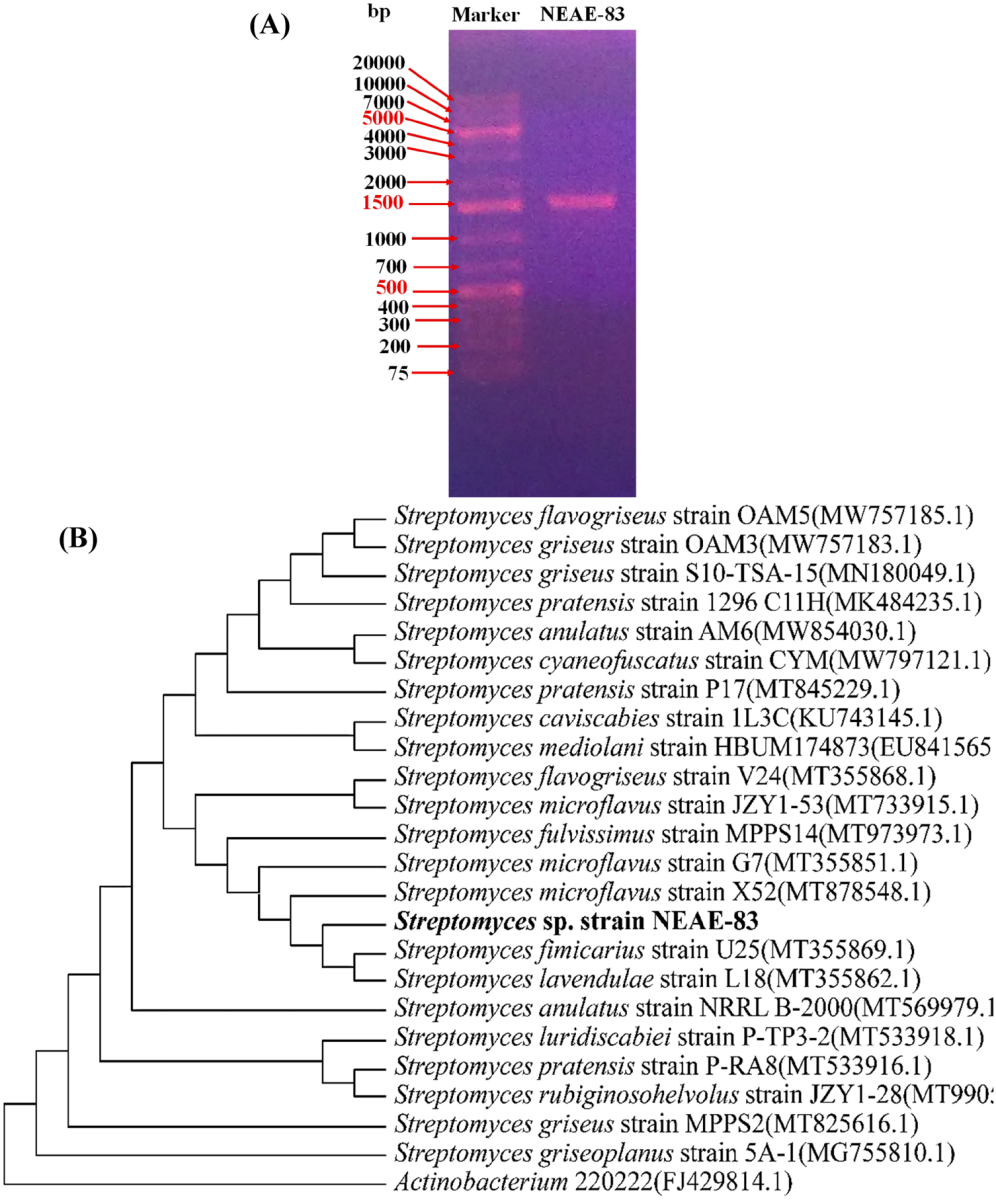
The outline of the electrophoresis of agarose gel of PCR product bands of the amplified 16S fragment (1214 bp) obtained from *Streptomyces* sp. strain NEAE-83 (MG384964) (**A**), and the neighbor-joining phylogenetic tree (**B**).

The phylogenetic neighbor-joining tree (Fig. 4B), constructed based on the sequence of 16S rRNA gene (1214 bp), shows the associations between *Streptomyces* sp. strain NEAE-83 and interrelated species of the genus *Streptomyces* on the GenBank. The analysis of the phylogenetic tree specified that *Streptomyces* sp. strain NEAE-83 falls into a clade together with *Streptomyces microflavus* strain X52 (GenBank/EMBL/DDBJ accession No. MT878548.1), *Streptomyces microflavus* strain G7 (GenBank/EMBL/DDBJ accession No. MT355851.1), *Streptomyces fulvissimus* strain MPPS14 (GenBank/EMBL/DDBJ accession No. MT973973.1), *Streptomyces fimicarius* strain U25 (GenBank/EMBL/DDBJ accession No. MT355869.1) and *Streptomyces lavendulae* strain L18 (GenBank/EMBL/ DDBJ accession No. MT355862.1).

According to the study of the 16S rRNA sequence of *Streptomyces* sp. strain NEAE-83, together with its morphological and physiological characteristics, *Streptomyces* sp. strain NEAE-83 was classified as *Streptomyces microflavus* strain NEAE-83*.* The strain was deposited in the Genbank, and the accession number was obtained as MG384964**.**

### Characterization of CNPs

Following the selection of *Streptomyces microflavus* strain NEAE-83, and the assurance of its efficacy in the bioconversion of bulk chitosan into CNPs, the latter was investigated through several characterization procedures.

### Electron microscopy investigation

**Figure 5:**
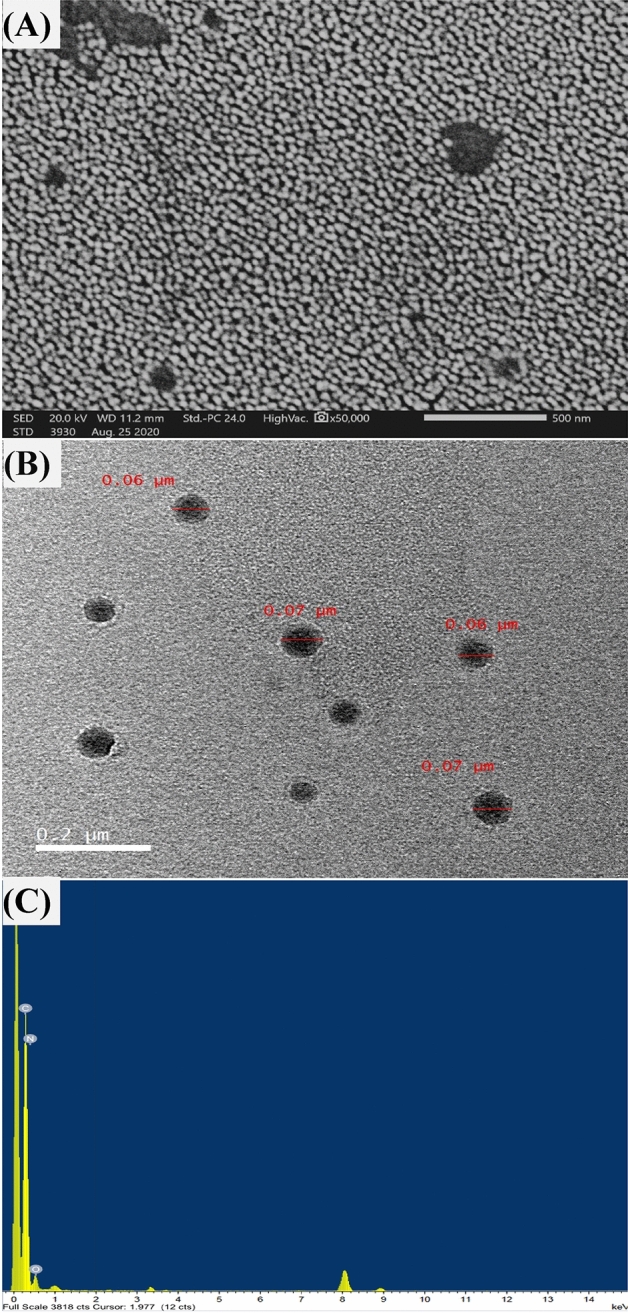
The biosynthesized chitosan nanoparticles using *Streptomyces microflavus* strain NEAE-83, as detected by the micrographs of SEM (**A**), TEM (**B**) and EDXS (**C**).

Investigation of CNPs via scanning electron microscopy (SEM) displayed details on the size and surface morphological construction. CNPs show spherical-like particles (Fig. 5A), with uniformity and homogeneity, without any visible agglomeration. The image of the morphological structure generated by transmission electron microscopy **(**TEM) displayed the size of CNPs at a range from 60 to 70 nm, without aggregation, and agglomeration (Fig. 5B). SEM and TEM were harmonized in CNPs identification. Both SEM and TEM are efficient tools for discovering nanoparticles, and widely accepted tools for the examination of morphological formation e.g., shape, size, and surface area^33^.

The spherical-like particles obtained in this study were in agreement with the spherical or oval shapes of most of the CNPs preparations^34,35^. Moreover, the noticed well-dispersed and entangled CNPs lead to the creation of a larger surface area, suggesting the suitable of CNPs for processes that depend on adsorption criteria^36^.

CNPs that have a small particle size (60–100 nm) and a large surface area could be essential for a wide variety of applications including fabrics^37^. The particle size of chitosan nanoparticles has a significant impact on the properties of these particles when they are used in pharmaceutical applications. A smaller particle size has the potential to encapsulate a greater quantity of therapeutic substances, improve the drug's stability and bioavailability, and make it possible for the drug to be administered for a longer period of time^38^. Chandrasekaran et al.^39^ proposed that the surface area increases as the particle size decreases. That might be the determining factor in the increased antibacterial activity of nanoparticles of small size.

In disagreement with bulk materials, the current CNPs image shows the non-agglomeration. Although several nanoparticles may have a high ionic force that causes aggregation and agglomeration in the aqueous phase, the CNPs synthesized by *Streptomyces microflavus* strain NEAE-83 showed no aggregation or agglomeration. There is a distinct variation between them. Aggregation describes toughly bonded or even fused particles whereas agglomeration reveals faintly bonded particles, however, even little agglomeration of CNPs may be accepted^40^.

### Energy-dispersive X-ray spectroscopy (EDXS)

The kinds, distributions, and concentrations of elements in CNPs were explored using EDXS via TEM (Fig. 5C). The EDXS spectra of CNPs approve the existence of the main elemental component of native chitosan (carbon, nitrogen, and oxygen). During the fabrication process, CNPs may be subjected to changes in the structure, so EDXS may help detect any variation. Where the internal shell of the atom is hit by a TEM electron ray, in which its electron(s) is displaced by another electron from the external shell to fill the empty position. This process leads to variation in energy in the form of X-ray, the intensity of the X-ray is straightforwardly connected to the concentration and is unique for every element^41^.

### Particle size analysis

**Figure 6:**
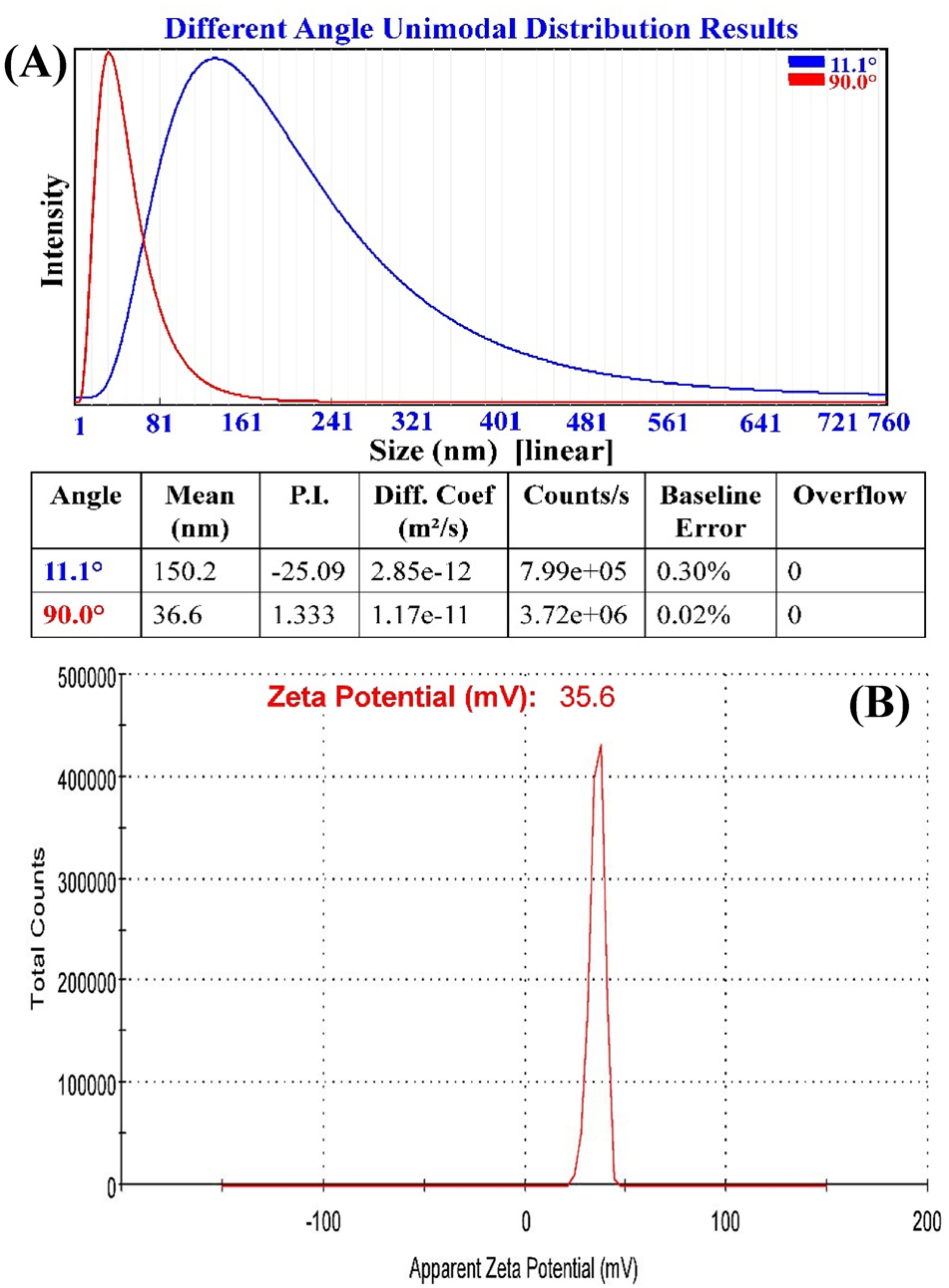
Analyses of chitosan nanoparticles with (**A**) PSA and (**B**) ζ-potential for bio-synthesized with *Streptomyces microflavus* strain NEAE-83.

The particle size distribution of chitosan nanoparticles suspension was investigated in this study using a particle size analyzer (PSA). The size distribution was determined at room temperature and included a wavelength range of 1 to 760 nm. The particle size analyzer measurement of a chitosan nanoparticles sample revealed a narrow and sharp peak at 36.6 nm diameter at θ =  90° and 150.2 nm at θ = 11.1° as shown in Fig. 6A. The sizes recorded by the particle size analyzer can only be taken as a relative value and cannot be compared with that determined by electron microscopy^42^. Electron microscopy is capable of detecting the geometric dimensions of the particles given by measuring the width of individual particles from the image, and determining their shape and surface structure (e.g. texture)^43,44^. Imaging was favored because of its high-resolution visualization of particles and the minimal effect of artifacts on size determination^44^.

### Zeta (ζ) potential analysis

The biosynthesized CNPs were investigated to find out ζ-potential value. The depicted ζ-potential (Fig. 6B) shows that the dispersal has a sole peak, representing brilliant uniformity of CNPs, which had a positive charge with ζ-potential of + 35.6 mV, which confirms the presence of surface protonated amine groups. The current ζ-potential value suggests good stability of the biofabricated CNPs.

The analysis of ζ-potential is a unique way of exploring the characteristics of colloidal dispersals. Zeta potential can have a significant impact on the stability of particles in suspension because of the electrostatic repulsion that exists between individual particles^45^. Yien et al.^46^ reported that the zeta potential of chitosan nanoparticles ranged from + 22 to + 55 mV and their inhibitory effect was influenced by particle size and zeta potential of chitosan nanoparticles. Kheiri et al.^47^ revealed that the zeta potentials of CNPs surfaces have a positive charge about of 45.6 mV. According to the findings of Khan et al.^48^, Raza and Anwar^49^ and Asal et al.^50^, the zeta potential on the surface of CNPs was determined to be + 31 ± 3.14, + 31.3 and + 31 ± 2.2 mV; respectively. In contrast, Qi et al.^51^ reported that the surfaces of chitosan nanoparticles had a positive charge of about 51 mV. On the other hand, Iswanti et al.^52^ reported that the surfaces of chitosan nanoparticles had a positive surface charge of + 3.3 ± 0.4 mV. Despite the fact that the suspension is physically stable, Manikandan and Sathiyabama^53^ mentioned that a zeta potential of at least ± 30 mV is necessary as a minimum for a NPs suspension to be stabilized principally by electrostatic repulsion. If the zeta potential is smaller than + 30 mV, this indicates that the CNPs have less stability due to lower electrostatic repulsion^54^. Another, the positive ζ-potential may be considered another advantage i.e., as antimicrobial agent. Positive ζ-potential of the particles enables ease of interacting with the negative charges on the cell membrane and DNA, then released into the cytoplasm^55^.

### Mapping analysis

**Figure 7:**
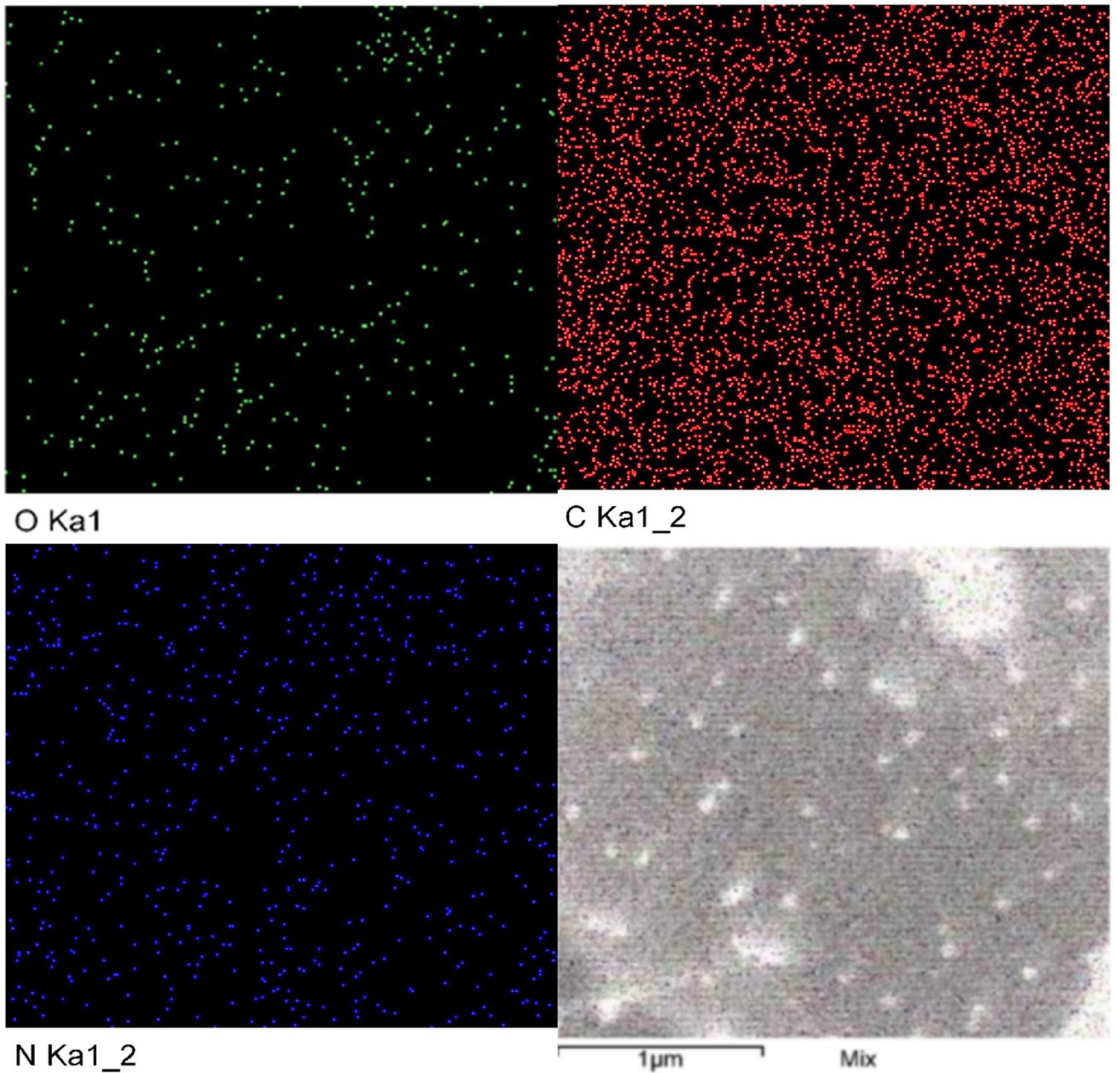
Mapping analysis of chitosan nanoparticles bio-synthesized using *Streptomyces microflavus* strain NEAE-83.

A mapping analysis was carried out to give a closer look at the behaviors of the CNPs community. The current mapping analysis of the individual responses of nanoparticles was carried out to investigate the pattern of locations and distribution of the elemental nanomaterial (Fig. 7). The data recovered from the electron microscopy show the whole image of the distribution of CNPs that distributed evenly. However, the individual O, C, and N elements of CNPs have the same pattern, where they are evenly scattered and distributed.

### Fourier transform infrared (FTIR) investigation

**Figure 8:**
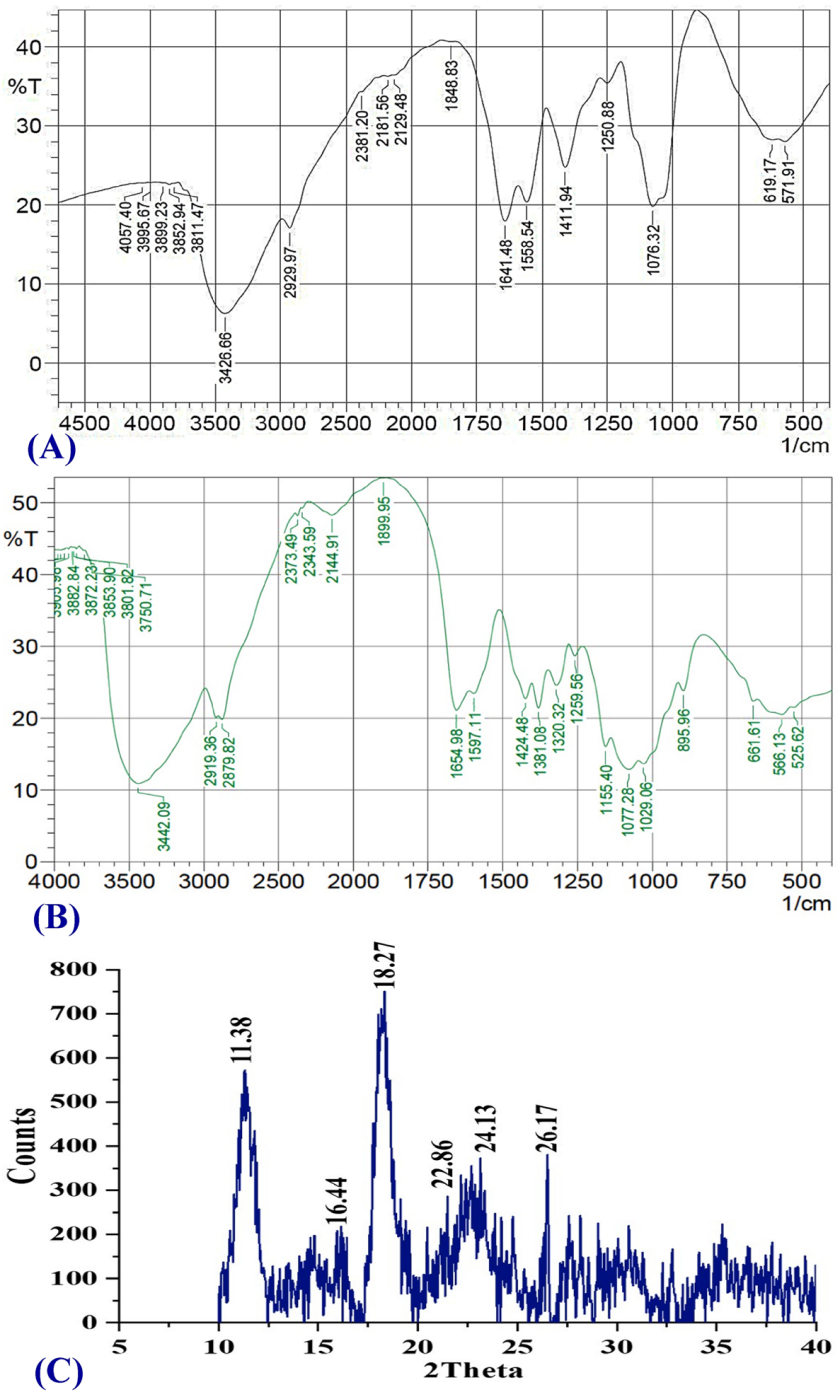
Examination of FTIR for the bio-synthesized chitosan nanoparticles (**A**), for chitosan standard (**B**) and XRD (**C**) of CNPs fabricated using *Streptomyces microflavus* strain NEAE-83.

FTIR analysis was carried out to detect the possible incidence of different binding functional groups with CNPs owing to the stabilization and reduction action of *Streptomyces microflavus* strain NEAE-83 supernatant. The FTIR spectrum of the bio-synthesized CNPs was analyzed and compared with the FTIR spectrum of a chitosan standard (Fig. 8A,B). The first group of bands appeared in the spectra between 4057 and 3750 cm^−1^, indicating the combination of functional groups of –NH_2_, –CH, C–C, and –OH.

Significant shifts of peaks in the spectrum of CNPs from peaks in the spectrum of the chitosan standard indicate a significant role of functional groups in the CNPs biosynthesis. The presence of bands at 3442 cm^−1^ in the chitosan standard sample indicates strong dimeric OH stretch^56^. Moreover, on the formation of nanoparticles, the hydroxyl (OH) stretching peak at wavelength 3442.09 cm^−1^ found in the spectrum of chitosan standard shifted to 3326.66 cm^−1^ which indicates the stretching vibrations of OH groups (Fig. 8A,B). A band at 3420 cm^−1^ is attributed to combined peaks of –OH and –NH_2_ groups stretching vibration in chitosan^57^. The aliphatic stretching group (CH and CH_2_) appeared at 2929 cm^−1^. At 2381–2129 cm^−1^ the stretching vibrations of C=C conjugated and C≡C has emerged.

Krishnaveni and Ragunathan^56,58^ reported that the bands at 1655 cm^−1^, and those at 1641, 1642 cm^−1^ indicate the presence of the amide I region. Further, the band at 1654.98 cm^−1^ (around 1655 cm^−1^) indicates the stretching vibration of type I amide. The intense peak of the amide I region shifted from 1654.98 cm^−1^ in the FTIR spectrum of chitosan standard to 1641.48 cm^−1^ in the FTIR spectrum of CNPs, indicating interactions between protonated amine groups of the chitosan standard with the components of the culture filtrate of *Streptomyces microflavus* strain NEAE-83. In chitosan nanoparticles, this peak is sharper and shifts toward 1641.48 cm^−1^ indicating increased interactions or bonding. So, the shifting of vibrations from higher to lower wave numbers reveals the formation of CNPs^57^.

The peak around 1558 cm^−1^ in the FTIR spectrum of CNPs is due to stretching vibrations of Amide II (CONH_2_)^57^. CNPs spectrum showed a band around 1412 cm^−1^ indicating aromatic C–C stretch^57^. In the FTIR spectrum of chitosan standard, Amide III region presence was indicated by a band at 1381 cm^−1^^57^. Bands at 1381, 1320 and 895 cm^−1^ disappeared in the FTIR spectrum after CNPs biosynthesis, revealing that these groups are involved in the CNPs biosynthesis by the culture filtrate of *Streptomyces microflavus* strain NEAE-83. Absorption in the wavenumber around 1076 cm^−1^ is produced from the stretch vibration of CO groups (COH and COC) in the oxygen bridge, arising from chitosan deacetylation. The small peaks located at the end of the FTIR spectra correspond to the wagging of the saccharide structure of chitosan^45,59^.

FTIR analysis declares the occurrence of capping groups to the surface of CNPs, which stabilizes the CNPs, as well as prevents their coagulation and/or aggregation in the colloidal phase.

### X-ray diffraction (XRD) analysis

XRD was utilized for exploring the crystallographic building of CNPs. The pattern of XRD is used in materials science as a fast and primary procedure for the phase detection of the crystallinity to give enough perspective on the unit aspects. As a consequence of this, XRD has been designated as a fingerprint for a specific substance. X-rays irradiation led to the various dispersion angles and intensities of the ray.

The XRD pattern of CNPs sample showed six distinctive peaks at 2*θ* which were at 11.38, 16.44, 18.27, 22.86, 24.13 and 26.17° (Fig. 8C) indicating a shift from the normal chitosan peaks. Rasaee et al.^59^ reported that the CNPs exhibited diffraction peaks at 2θ = 10° (weak diffraction peak) and 20° (strong diffraction peak), revealing that the chitosan had a high degree of crystallinity. XRD spectrum of CNPs indicates the peak shift from the normal peaks of natural chitosan, which corresponds to the reduction of crystallinity and the increased amorphous structure. The amorphous structure of CNPs contributes to an increased capacity for sorption^45,60^.

### CNPs' thermal characteristics

The thermoanalytical properties of the bacterially generated CNPs were tested throughout a controlled temperature range, employing two main ways: thermogravimetric analysis (TGA) and differential scanning calorimetry (DSC). Since the thermoanalytical properties of nanoparticles participate in chemical processes. The primary distinction between TGA and DSC is the technique used to quantify the changes in samples caused by heat^35,60,61^.

**Figure 9:**
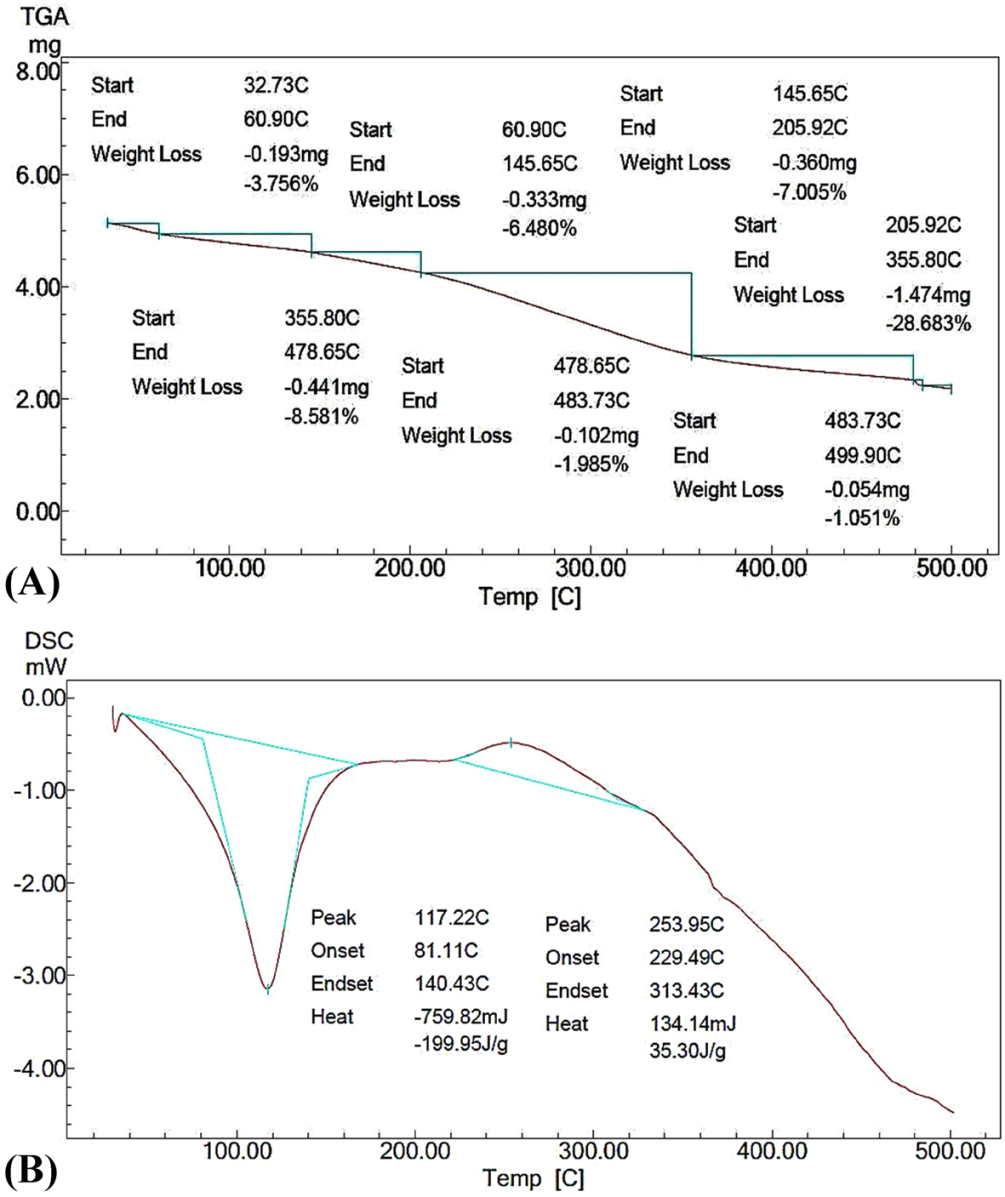
Chromatograms of TGA (**A**) and DSC (**B**) investigation of CNPs fabricated using *Streptomyces microflavus* strain NEAE-83.

TGA was accomplished to track the thermal behavior of CNPs as a consequence of a continual fluctuation in the heating ratio (from 25 to 500 °C) to explore mass fluctuation in physical and chemical features of CNPs with the change in temperature (Fig. 9A). TGA showed that from 32.73 up to 60.90 °C, a quick initial mass dropping (− 3.756%) can easily be detected. At higher temperatures, weight losses of CNPs were observed with multistage decomposition. The temperature range of 205.92–355.80 °C recorded the highest weight loss (− 28.683%), which was 1.474 mg, while the lowest weight loss (− 1.051%) was measured over a range of 483.73–499.90 °C.

TGA determines differential thermal analysis by determining the relationship between the alteration in the amount of the sample and different temperatures, or as a fixed temperature at a time and constant loss of mass^35,60,61^. Commonly, the temperature at the start of TGA causes dryness of CNPs and can easily cause a quick drop mass of CNPs due to the loss of water-bound located in the two polar groups, this drying change does not result in chemical reactions or lead to any structural alterations^60^. Another reason for the initial weight drop could be the breakdown of volatile units, dehydration of the saccharide rings, and depolymerization^36^. Later, as the temperature rises, sequential losses in CNPs weight may occur owing to sublimation and/or evaporation. Nevertheless, at higher temperatures, a multistage decomposition owing to the thermal degradation of CNPs could be observed in the form of step-like patterns^61^.

Even though the phases of TGA did not interfere during dynamic behavior as seen in the graph, there is a possible unseen decomposition interference, requiring either a much lower rating of heat or using the stepwise (step-by-step) TGA approaches. As a result, TGA alone may not be enough to detect the destructed products; consequently, DSC is frequently essential in addition to TGA to ascertain the presence of intermediate destructed products^35^.

The thermo-analytical investigation of the DSC was conducted at various rates of heating to clarify the amount (positively or negatively) of variation in the heat flow of CNPs as a result of temperature in the occurrence of the solvent as a reference. Thermo-analytical information was gathered for both CNPs and solvent reference to produce a phase schema (Fig. 9B). With the change in the thermodynamic system, two-phase transitions of CNPs were detected with certain wide endothermic peaks. One peak at 117.22 °C was identified through the range of 81–140 °C, requiring − 199.95 J of heat per gram of CNPs. At 253.95 °C, another peak emerged, resulting in 35.30 J of heat per gram of CNPs. At 229 °C a sole glass transition phase of the CNPs was detected.

The DSC was used to calculate the required or released heat flow versus the change of temperature at a specific time. The thermal analysis displayed some weight loss in the advance stages due to the CNPs decomposition. However, the nanoparticles produced by *Streptomyces microflavus* strain NEAE-83 did not completely disintegrate at high temperatures (500 °C) and demonstrated some constancy in the structure of the polysaccharide, which could be attributed to the elevated crosslinking feature of the CNPs, which procedures a sturdier and firmer network of the hydrogel^36,60,61^.

The phase transitions of the thermal degradation mechanism for every single step were highlighted. The DSC settings are planned to enable the linear holding temperature of a sample as a function of time. So, the physical phenomena of a material can be tracked, such as thermal constancy, purity, and glass transition^35^.

The first endothermic peak was generated at a low temperature owing to the elimination of water. Then a broad endothermic peak emerged later at 253.95 °C due to the breaking of CNPs cross-linkage. As well, one single glass transition (the phase at which material drives from a hard brittle state into a soft rubbery one) was found in the DSC heating curves at high temperature (229 °C), this is because of the existence of an extreme thermal constancy of the crosslinking, demonstrating the consistency of the CNPs^36^. The reduction in crystallinity of CNPs after transformation may be owing to alterations in chitosan's solid-state building caused by crosslinking, and thus CNP breakdown happens above 300 °C^60^.

All previous specifications provide a precise perspective for characterizing CNPs and came into agreement with each other and previous works. Furthermore, the currently proposed microbial method is thought to be ideal for producing high-quality CNPs.

### Optimization of CNPs biosynthesis by *Streptomyces microflavus* strain NEAE-83

Finding out the best settings for extreme biosynthesis of CNPs by *Streptomyces microflavus* strain NEAE-83 is the prime aim of the trial optimization policy. The bio-fabrication process by *Streptomyces microflavus* strain NEAE-83 was optimized and modeled based on the advanced technique of RSM, then an artificial intelligence approach was applied. The prediction models by both approaches were generated and compared, as an unprecedented procedure in this field.

### Central composite face-centered design (CCFCD)

The statistical modulating approach of RSM was used to set up a four-factors matrix of CCFCD to explore the maximum combination as well as the effects of individual, interaction, and quadratic variables of the tested independent factors on CNPs biosynthesis by *Streptomyces microflavus* strain NEAE-83. The design matrix and the levels (actual and coded) of the four variables tested, as well as the investigational and predicted CNPs values and their residual errors are presented in Table 2. The trial runs showed various degrees of responses that reached up to 9.41 mg/mL (run  27).

**Table 2:** Four-factors CCFCD matrix with the corresponding actual and predicted (CCFCD and ANN) values of chitosan nanoparticles biosynthesized by *Streptomyces microflavus* strain NEAE-83.

Std	Run	Coded levels of the independent variables				Chitosan nanoparticles biosynthesis (mg/mL)				
		X_1_	X_2_	X_3_	X_4_	Actual	CCFCD		ANN	
							Predicted	Residuals	Predicted	Residuals
30	1	0	0	0	0	4.09	4.08	0.01	4.10	− 0.01
20	2	0	1	0	0	3.5	3.55	− 0.05	3.60	− 0.10
9	3	− 1	− 1	− 1	1	5.11	5.29	− 0.18	5.12	− 0.01
15	4	− 1	1	1	1	2.81	2.77	0.04	2.83	− 0.02
17	5*	− 1	0	0	0	3.43	3.31	0.12	3.22	0.21
24	6	0	0	0	1	4.38	4.29	0.09	4.44	− 0.06
28	7	0	0	0	0	4.19	4.08	0.11	4.10	0.09
2	8	1	− 1	− 1	− 1	4.41	4.47	− 0.06	4.40	0.01
7	9	− 1	1	1	− 1	3.94	3.90	0.04	3.94	0.00
13	10	− 1	− 1	1	1	5.3	5.15	0.15	5.27	0.03
14	11	1	− 1	1	1	6.73	6.75	− 0.02	6.67	0.06
19	12	0	− 1	0	0	4.03	4.13	− 0.10	4.08	− 0.05
1	13	− 1	− 1	− 1	− 1	2.66	2.53	0.13	2.68	− 0.02
26	14	0	0	0	0	4.31	4.08	0.23	4.10	0.21
29	15*	0	0	0	0	4.25	4.08	0.17	4.10	0.15
16	16	1	1	1	1	4.19	4.26	− 0.07	4.20	− 0.01
18	17	1	0	0	0	4.77	5.03	− 0.26	4.85	− 0.08
10	18*	1	− 1	− 1	1	3.83	3.81	0.02	4.02	− 0.19
23	19	0	0	0	− 1	4.95	5.18	− 0.23	4.96	− 0.01
8	20	1	1	1	− 1	8.97	8.81	0.16	8.95	0.02
21	21*	0	0	− 1	0	3.84	3.63	0.21	3.86	− 0.02
12	22*	1	1	− 1	1	3.06	2.94	0.12	3.18	− 0.12
11	23	− 1	1	− 1	1	4.37	4.52	− 0.15	4.33	0.04
4	24*	1	1	− 1	− 1	5.59	5.68	− 0.09	5.62	− 0.03
22	25	0	0	1	0	4.77	5.13	− 0.36	4.82	− 0.05
3	26	− 1	1	− 1	− 1	3.84	3.84	0.00	3.81	0.03
6	27	1	− 1	1	− 1	9.41	9.21	0.20	9.37	0.04
5	28	− 1	− 1	1	− 1	4.05	4.19	− 0.14	4.08	− 0.03
27	29	0	0	0	0	4.21	4.08	0.13	4.10	0.11
25	30	0	0	0	0	3.87	4.08	− 0.21	4.10	− 0.23

Variable	Code	Low (− 1)	Center (0)	High (1)						
Initial pH	X_1_	4.5	5.0	5.5						
Chitosan concentration (%)	X_2_	1.0	1.5	2.0						
Temperature (°C)	X_3_	30	35	40						
Incubation period (h)	X_4_	12	24	36						

*Six runs were chosen at random for validating the ANN model, while the remaining 24 runs were used for training.

Based on the data obtained from CCFCD, the linear, two-factor interaction, and quadratic models were compared to choose the best fit model (Table 3). Comparison of the model statistics such as lack of fit, and the sum of squares concluded that the quadratic model was the ideal choice, recording insignificant lack of fit error for the probability value (*P* > 0.05) whereas, the model *P*-value was significant. Both the sum of squares of prediction error (PRESS) and the standard deviation were lower in comparison with the other models. Finally, the quadratic model had higher values of determination coefficient (R^2^), adjusted-R^2^, and predicted-R^2^. Therefore, the quadratic model was chosen for the molding CNPs biofabrication process. When using the quadratic model that was created using the CCFCD, the predicted values of CNPs were found to be in line with the actual ones (Table 2).

**Table 3:** Fitting’s summary of face-cantered central composite design based on the experimental data.

Source	Sum of squares	Degrees of freedom	Mean square	*F-*value	*P-*value
**Lack of fit tests**					
Linear	36.41	20	1.82	74.17	< 0.0001*
2FI	5.09	14	0.36	14.82	0.0038*
Quadratic	0.57	10	0.06	2.34	0.1801
Pure Error	0.12	5	0.02		
**The sequential model sum of squares**					
Linear versus Mean	28.45	4	7.11	4.87	0.0048*
2FI versus Linear	31.32	6	5.22	19.01	< 0.0001*
Quadratic vis 2FI	4.52	4	1.13	24.29	< 0.0001*
Residual	0.20	7	0.03		

Source	Standard deviation	R^2^	Adjusted R^2^	Predicted R^2^	PRESS
**Model summary fittings**					
Linear	1.21	0.4378	0.3479	0.018	63.82
2FI	0.52	0.9197	0.8775	0.7214	18.11
Quadratic	0.22	0.9893	0.9793	0.9495	3.28

*Significant values, R^2^: coefficient of determination, PRESS: prediction error sum of squares, 2FI: two factors interaction.

The quadratic CCFCD model was selected based on the effectiveness of each model. The lower *P*-value (< 0.05) was reliably significant for the modeling CNPs biofabrication process. Another important choice criterion is R^2^. The R^2^ value indicates the extent to which the experimental parameters can explain the observed response values^62^. If R^2^ is 0.9 or greater, the model is regarded as adequate and more precise for predicting the response^63,64^. Generally, the closer to 1, the greater the modeling capacity of the data^18^. The present quadratic model has R^2^, adjusted R^2^, and predicted R^2^ values that are all actually close to one. The change in the CNPs experimental response results in the variation in the amount of a factor(s) is known as the R^2^ value, which is always ranged from zero to 1 for all types of R^2^^62^. Surprisingly, regardless of the significance of the factors, raising the number of predictors (factors) leads to a constant increase in the R^2^ value. As a result, the adjusted R^2^ is a modified version of R^2^ that takes into account the number of model factors. In contrast to R^2^, the adjusted R^2^ changed wisely when adding additional factor(s) to the model. As a result, adjusted R^2^ is a better indicator than R^2^ for determining model fitness. Finally, the predicted R^2^ is employed to evaluate the model's predictive potential, such as predicting the CNPs value at new untested levels of the factors. The values of predicted-R^2^ and Adjusted-R^2^ should be within 20% of each other, indicating that the model is very significant and accurate, as well as a reasonable agreement between them^65^. The predicted values of CNPs calculated based on the quadratic model of CCFCD were very closest to those of the experimental ones; consequently, the residuals or errors were low, representing another evidence of the accuracy of the model. Consequently, the linear and the two factors interaction models were omitted, and the quadratic model was chosen as the top-fitted one for CNPs bioprocessing by *Streptomyces microflavus* strain NEAE-83.

### Coefficients and multiple regression analysis

The data recovered from the CCFCD were exposed to both multi-regression and variance analysis (ANOVA) (Table 4). The overall model performed significantly at *P*-value < 0.05. All the model terms, including the effects of quadratic, interaction, and linear terms tracked a similar significant tendency, except the two factors interaction of the initial pH and chitosan concentration, as well as the quadratic effect of both initial pH and chitosan concentration. The lack-of-fit error of the model did not reach the significance threshold, recording a higher *P*-value of 0.1801. Furthermore, the model standard deviation, coefficient of variation, and adequate precision exhibited decent performance.

**Table 4:** Variance analysis and coefficient estimate for the experimental data of chitosan nanoparticles biosynthesis using *Streptomyces microflavus* strain NEAE-83 obtained by the matrix of CCFCD.

Source of variance	Analysis of variance					Coefficient estimate
	Degrees of freedom	Sum of square	Mean of square	*F*-value	*P*-value	
Overall model	14	64.29	4.59	98.76	< 0.0001*	4.08
**Linear**						
X_1_	1	13.26	13.26	285.19	< 0.0001*	0.86
X_2_	1	1.54	1.54	33.06	< 0.0001*	− 0.29
X_3_	1	10.07	10.07	216.45	< 0.0001*	0.75
X_4_	1	3.59	3.59	77.23	< 0.0001*	− 0.45
**Interaction**						
X_1_ X_2_	1	0.01	0.01	0.23	0.6414	− 0.03
X_1_ X_3_	1	9.44	9.44	203.02	< 0.0001*	0.77
X_1_ X_4_	1	11.68	11.68	251.17	< 0.0001*	− 0.85
X_2_ X_3_	1	2.58	2.58	55.57	< 0.0001*	− 0.40
X_2_ X_4_	1	4.36	4.36	93.71	< 0.0001*	− 0.52
X_3_ X_4_	1	3.25	3.25	69.87	< 0.0001*	− 0.45
**Square**						
X1^2^	1	0.02	0.02	0.47	0.5039	0.09
X_2_^2^	1	0.15	0.15	3.30	0.0895	− 0.24
X_3_^2^	1	0.23	0.23	4.91	0.0426*	0.30
X_4_^2^	1	1.12	1.12	24.03	0.0002*	0.66
**Error**						
Lack-of-fit	10	0.57	0.06	2.34	0.1801	
Pure error	5	0.12	0.02	98.76		
R^2^		0.9893	Standard deviation			0.22
Adjusted R^2^		0.9793	Mean			4.56
Predicted R^2^		0.9495	C.V. %			4.73
Adequate precision		43.79	PRESS			3.28

*Significant of probability level < 0.05, R^2^: determination coefficient, *F*: Fisher's test, C.V: Variation coefficient, PRESS: the prediction error sum of squares, X_1_; initial pH, X_2_; chitosan concentration (%), X_3_; temperature (°C), X_4_; incubation period (h).

The low *P*-value, together with the high *F*-value and the nonsignificant lack-of-fit indicated the significance of the suggested overall model. The *P*-value was used as a diagnosing tool for measuring the significance of both the model and the factors. The process variables with *P*-values ≤ 0.05 were judged to have significant effects on the response^66^. Also, most of the tested terms are significant (*P* < 0.05), representing the importance of the tested parameters for CNPs bio-fabrication, and further suggesting that the variables, with their levels, and CCFCD, are well specified and perform optimally for the microbial fabrication of CNPs.

The signal-to-noise ratio is measured by the adequate precision value; a value greater than 4 is desired and indicates a strong model fit^66^. The adequate precision ratio is greater than 4, indicating the fruitful model design space to maximize CNPs biosynthesis at the various tested ranges of the factors. Another element of trustworthiness was the minor coefficient of variation% (C.V.%) value, which is desirable for the model's reliability. The very low value of C.V.% indicates a greater precision of the performed experiments^63^. Furthermore, the three types of R^2^ are adequately high to support the significance of both the model and its variables. Both adjusted R^2^ and predicted R^2^ were in harmony. To be in reasonable agreement, both types of R^2^ should be no more than 20% of each other^18,67^, indicating high compatibility among trial and predicted values of biosynthesized CNPs and further signifying the reasonable model's predictive ability within the design space. Furthermore, the coefficient estimate revealed a variety of positive and negative effects. The negative coefficients of some model terms indicate the antagonistic impact at higher concentrations^16^, such a variable has an on the microbial construction of CNPs. Whereas a positive coefficient value designates a cooperative impact, and the high level of the variable(s) increases CNPs biosynthesis within the design area.

### Checking the model adequacy

**Figure 10:**
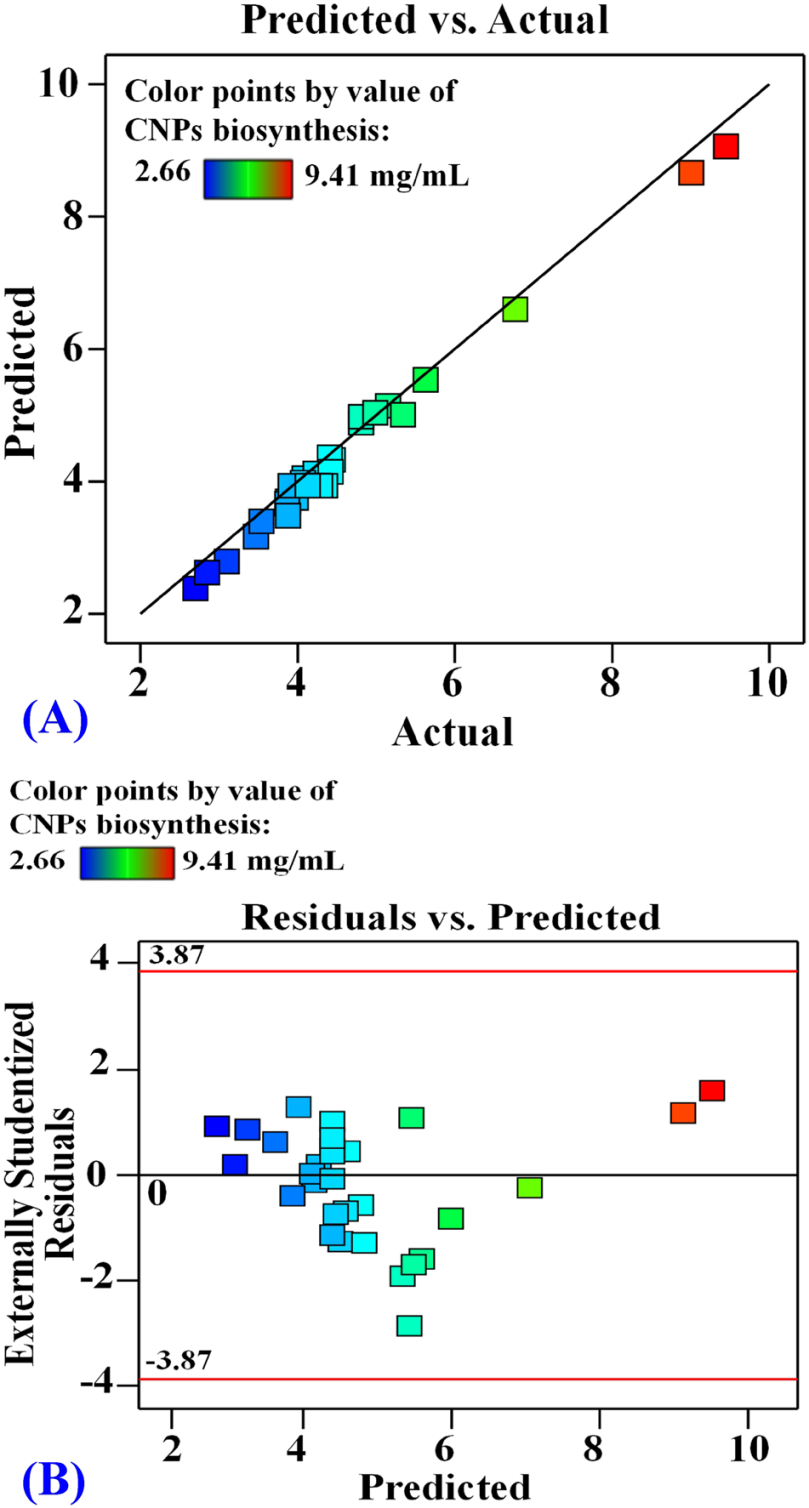
The predicted versus actual (**A**), and (**B**) the residuals versus predicted values of chitosan nanoparticles biosynthesis using *Streptomyces microflavus* strain NEAE-83.

To determine the model's suitability, the adequacy was investigated by plotting the prediction versus the actual points (Fig. 10A). The points of regression analysis are located much nearer to the perfect prediction line and display better fitting of the model predicted values and the experimental results, which confirms the accuracy of the model^5^.

Similarly, the externally studentized residual values of the model versus the predicted one were plotted (Fig. 10B). The graph shows an equivalent scattering of the residual points around the 0-axis, what is more, the residual points were found to be near the 0-axis without linearity. The graph showed that the externally studentized residual values scatter randomly around the 0-axis, suggesting that the variance of the experimental results is constant for all values^68^. This distribution pattern is sufficiently perfect to support the CCFCD model's applicability. Accordingly, the variance of CNPs data and the biosynthesis process were independent, demonstrating the model's adequacy and generalizability. As a result, these two sufficiency tests validate the design and data points as well.

### Dual relationship of the investigated factors

**Figure 11:**
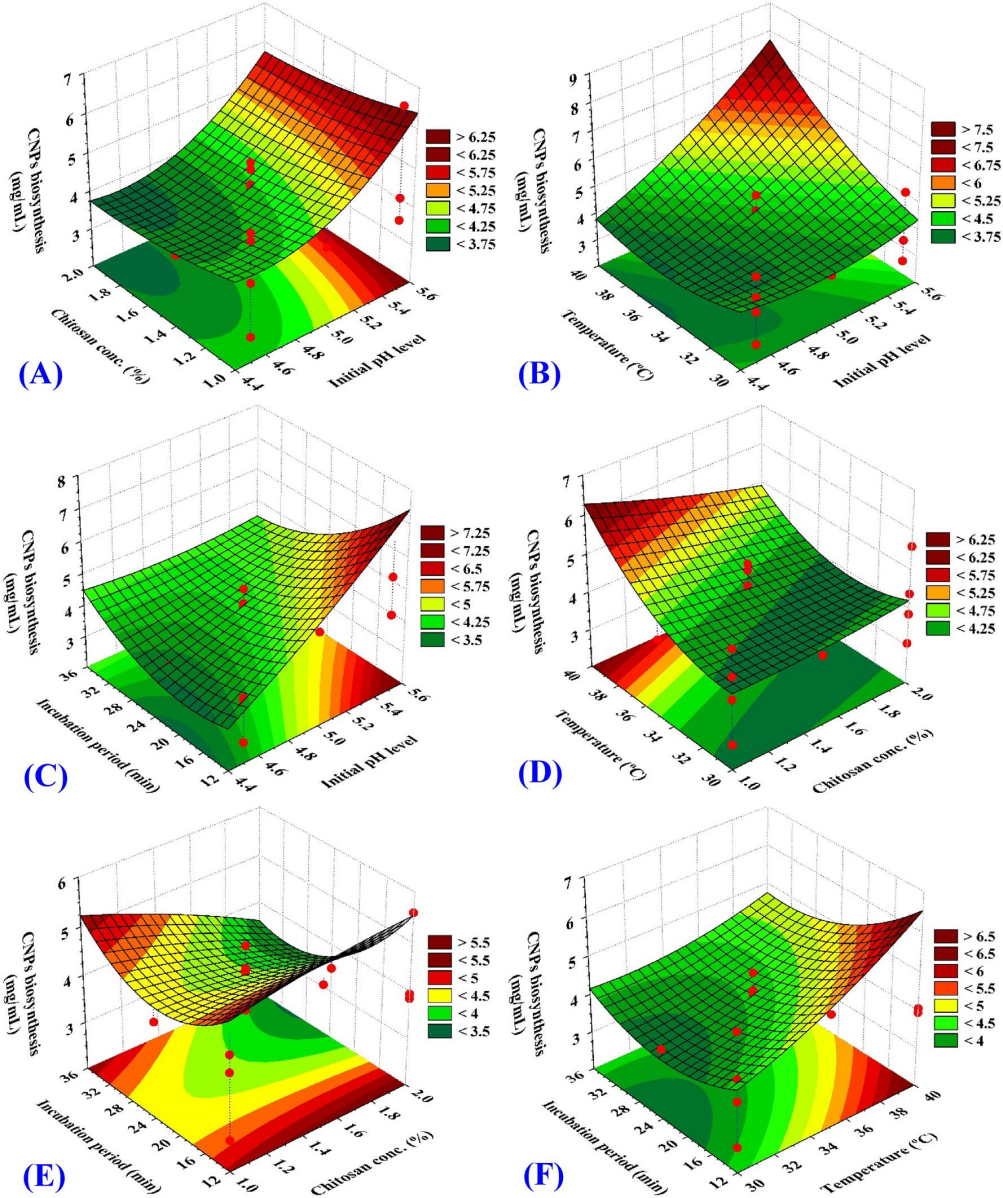
Three-dimensional surface plot of the interactive impact of each pair of factors for chitosan nanoparticles biosynthesis by *Streptomyces microflavus* strain NEAE-83. The other factors were kept at the center level.

In order to investigate the dual impact of every pair of the factors on CNPs biosynthesis using *Streptomyces microflavus* strain NEAE-83, the plots of the three-dimensional (3D) surface of the four independent factors were constructed (Fig. 11). The maximum CNPs biosynthesis was situated at the low chitosan concentration with a high pH value (Fig. 11A), the high incubation temperature with high initial pH (Fig. 11B), high initial pH with low incubation period (Fig. 11C), low chitosan concentration with high incubation temperature (Fig. 11D), low chitosan concentration with low incubation period (Fig. 11E), and low incubation period with high incubation temperature (Fig. 11F), Out of these ranges, CNPs biosynthesis declined sharply. In the analysis of the 3D plots, a tight relationship between every pair of the tested factors could be noticed. This means that the factors’ ranges were wisely chosen, and the model is best appropriate for the design. Accordingly, the greatest combination of the tested in terms of the coded level was estimated based on the next CCFCD model's prediction function:

$$\begin{aligned} & {\text{CNPs biosynthesis }}\left( {{\text{mg}}/{\text{mL}}} \right) = { 4}.0{8} + 0.{86}\left( {{\text{X}}_{{1}} } \right) \, - 0.{29 }\left( {{\text{X}}_{{2}} } \right) \, + \, 0.{75 }\left( {{\text{X}}_{{3}} } \right) \, \\ & \quad \quad - \, 0.{45 }\left( {{\text{X}}_{{4}} } \right) \, - \, 0.0{3 }\left( {{\text{X}}_{{1}} {\text{X}}_{{2}} } \right) \, + \, 0.{77 }\left( {{\text{X}}_{{1}} {\text{X}}_{{3}} } \right) \, - \, 0.{85 }\left( {{\text{X}}_{{1}} {\text{X}}_{{4}} } \right) \, - \, 0.{4}0{ }\left( {{\text{X}}_{{2}} {\text{X}}_{{3}} } \right) \, \\ & \quad \quad - \, 0.{52 }\left( {{\text{X}}_{{2}} {\text{X}}_{{4}} } \right) \, - 0.{45}{ }\left( {{\text{X}}_{{3}} {\text{X}}_{{4}} } \right) \, + \, 0.0{9 }\left( {{\text{X}}_{{1}} } \right)^{{2}} - \, 0.{24 }\left( {{\text{X}}_{{2}} } \right)^{{2}} + \, 0.{30 }\left( {{\text{X}}_{{3}} } \right)^{{2}} + \, 0.{66 }\left( {{\text{X}}_{{4}} } \right)^{{2}} \\ \end{aligned}$$

where X_1_; initial pH, X_2_; chitosan concentration (%), X_3_; temperature (°C), X_4_; incubation period (h).

### Modeling CNPs biosynthesis by ANN

The CCFCD data were subjected to more advanced modeling procedures i.e., the artificial intelligence-based approach (Table 2) through machine learning. A predictive ANN model was developed. A fully connected multilayer feed-forward ANN platform was created for modeling the CNPs biosynthesis by the candidate actinomycete.

Despite artificial intelligence has made its road into recent scientific research, no work was reported on the modulation of biofabrication of CNPs by ANN. This is the first work to demonstrate the feasibility of this type of modeling. As a result, artificial intelligence was offered as a possible model for the experimental CCFCD data.

The finest architectural structure was established, numerous hidden layers and neurons within each hidden layer were tested at numerous patterns of ANN-specific factors. The machine learning continued until the error values i.e., mean absolute deviation (MAD), the sum of squared errors (SSE), and root mean square error (RMSE), reached their minimal, complemented by the uppermost value of R^2^, for both training and validation points (Table 5). Numerous trials learning, 5000 tours each, were performed, accordingly, the optimum ANN parameters were found to be at a learning rate of 0.1, engaging the procedure of holdback at 0.2 proportion. The activation function operated at the hidden layers’ nodes was the hyperbolic tangent sigmoid (NTanH).

**Table 5:** The performance of CCFCD and ANN models for CNPs biosynthesis by *Streptomyces microflavus* strain NEAE-83.

Model	R^2^	RMSE	MAD	Frequency
**Training statistics**				
CCFCD	0.9900	0.1565	0.1299	24
ANN	0.9964	0.0933	0.0666	24
**Validation statistics**				
CCFCD	0.9714	0.1354	0.1212	6
ANN	0.9810	0.1104	0.0869	6

Statistics		CCFCD	ANN	Frequency
**Overall model performance**				
R^2^		0.9893	0.9957	30
RMSE		0.1525	0.0970	30
MAD		0.1281	0.0707	30
SSE		0.6975	0.2748	30

RMSE; root mean squared error, MAD; mean absolute deviation, SSE; the sum of squares error.

**Figure 12:**
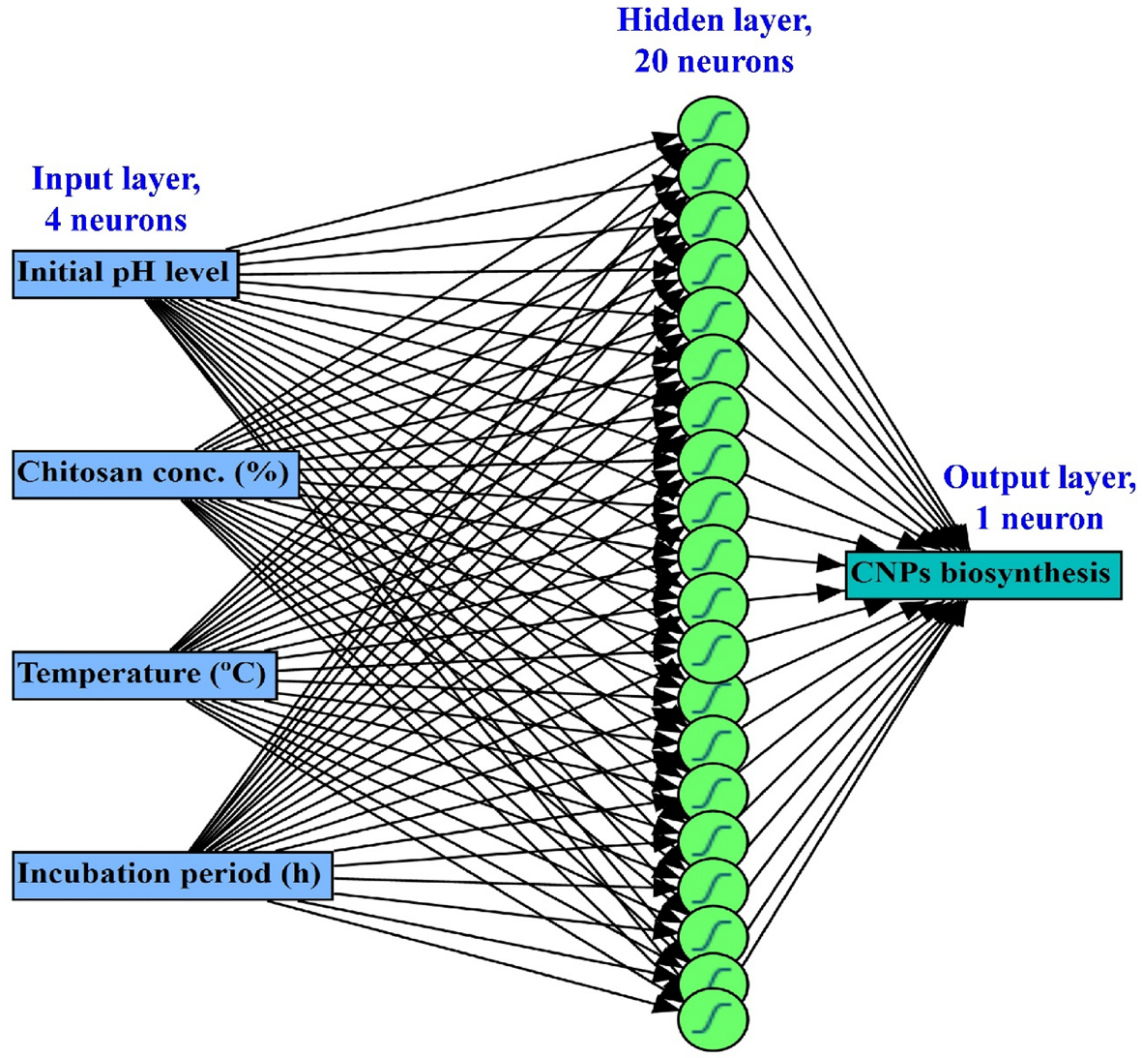
The final artificial neural network of CNPs biosynthesis by *Streptomyces microflavus* strain NEAE-83. The architecture scheme shows an input layer (4 neurons), a hidden layer (20 neurons), and an output layer with a single neuron.

The topology of ANN under such conditions was found to perform better when having one input layer comprised of the four-neurons (independent factors), one output layer of one-neuron (CNPs biosynthesis), and one in-between hidden layer with 20 neurons; (NTanH (20). Accordingly, the best architectural structure of the ANN topology was generated as 4-20-1 (Fig. 12).

The ANN is flexible enough to generate efficient and accurate models from any kind of response surface, using adequate hidden layers and nodes. The ANN platform employs an algorithm for machine learning that predicts response variables utilizing a supple function. The activation function helps the ANN learn weights and maps the complex nonlinear relationship, even if there is no apparent relation between input(s) and output(s) variables, that is why ANN can predict and fit data very well by modeling different response surfaces using the suitable architecture, and capable of learning any nonlinear function in flexibility and accuracy way^20,21,69^.

The current neural network platform (4-20-1) employs an algorithm of machine learning, named a fully connected multilayer perceptron. It is worth mentioning that ANN has an intermediate layer rather instead of a direct path between the input factors and the output CNPs variable.

The intermediate (hidden) layers play a central role in managing a unique correlation between inputs and outputs, instead of the direct path; therefore, ANN is considered an exceptional predictor when the form of relationship between the response(s) and the inputs is not required or recognizable^20,21,69^. The ANN activation function employed in the hidden layer nodes is in charge of discovering such linear combinations of the individual variables, without designating the relationship between both the input(s) and the response^20,21,69^.

### Evaluation of ANN model

Built on such a model, the predicted values of ANN for each experimental data point were estimated (Table 2). Consequently, ANN predicted values show more sensible harmony with the trial ones, and, and their residuals displayed lesser values than those gotten by the CCFCD model.

**Figure 13:**
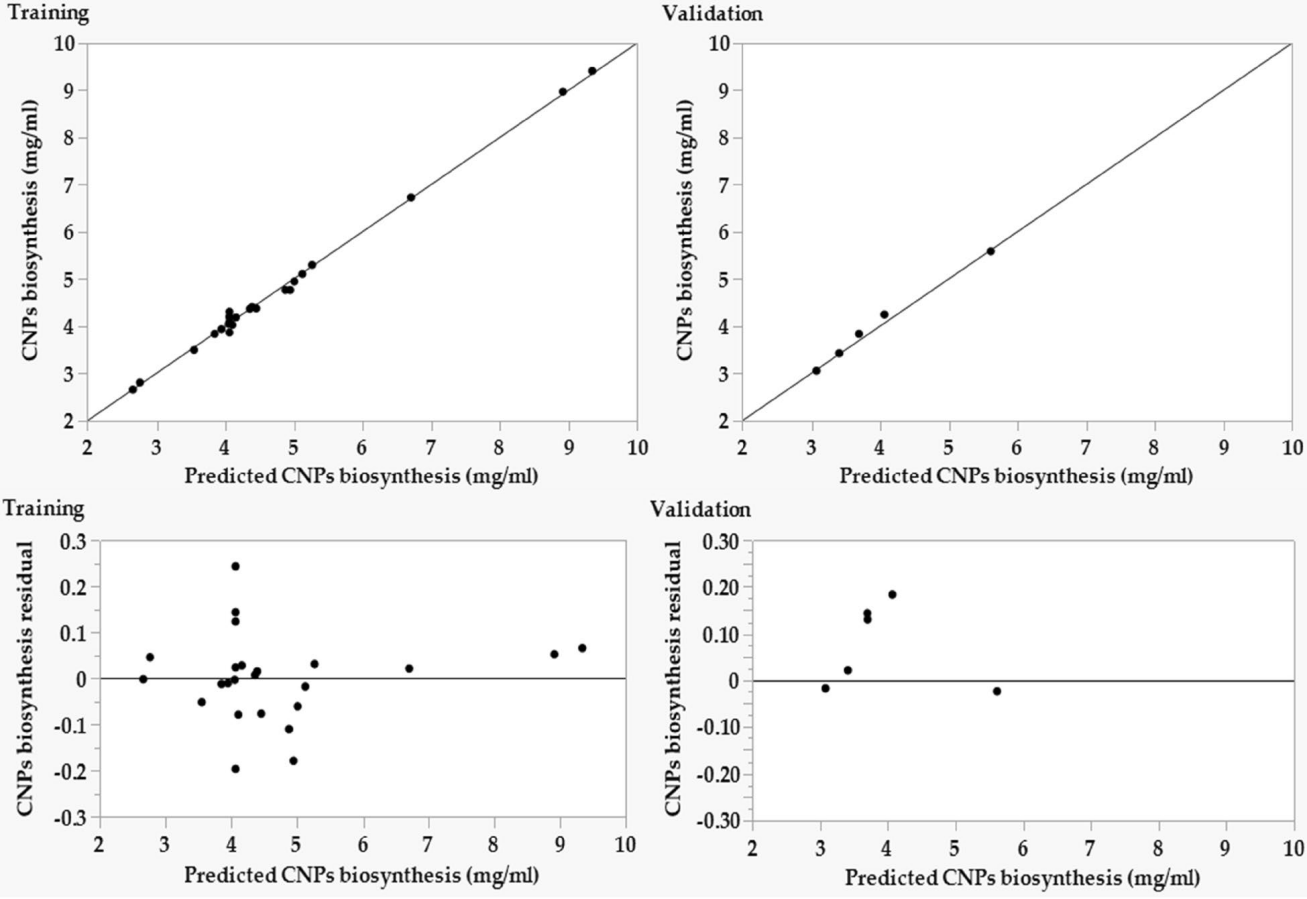
Chitosan nanoparticles biosynthesis versus the ANN predicted values and the residuals values using *Streptomyces microflavus* strain NEAE-83 for training and validation processes.

The predicted values of ANN were drawn against the actual values (Fig. 13). The analysis of linear regression demonstrates improved fitting points in relation to the actual ones. The points are located nearer to the line of the perfect forecast for both the training and validation process, endorsing the suitability of the model.

The residuals analysis plot shows a scattering of the residual data above and below the regression line. The residual analysis showed also a normal scattering of the residuals. This pattern is sufficiently perfect to support the ANN model's appropriateness.

### Comparison of CCFCD and ANN models

In comparison with the CCFCD model, the ANN model has high predictive points with lower residuals (Table 2), where the linear regression, and residual analysis display that the ANN model points (Fig. 13) are located much nearer to the slope of the best forecast than the points of CCFCD model (Fig. 10). This concludes that the ANN can fit the real investigational data precisely with a higher predictive capacity. Therefore, the generalization capability of the ANN model surpasses the CCFCD model.

The model's accuracy to predict microbial CNPs biosynthesis by both CCFCD and ANN was further compared with a collection of statistical parameters that were estimated for the overall model, as well as, for training and validation groups of CCFCD and ANN (Table 5). The R^2^ values of the ANN were high for both groups than the CCFCD model. In contrast to CCFCD, RMSE and MAD recorded lower values for ANN. The same trend was observed for the overall model i.e., greater R^2^ value and inferior RMSE, MAD, and SSE of ANN in contrasted to CCFCD, deducing the more confidence of ANN model than CCFCD.

As discussed earlier, R^2^ is used to determine the association between the CNPs response and projected values; thus, a bigger value in ANN designates an extra significant association between the two datasets than is reported in CCFCD. RMSE is engaged in regression assessment to validate trial results since a lesser value denotes that the data are aggregated around the area of best fit. MAD is another figure that signifies the average spreading of data around the mean. A minor MAD value infers a reduced spread of data around the mean. The value of SSE is an extra goodness-of-fit test, which is utilized to calculate the total divergence of the actual values from the fitted ones; a lower value designates that the model is appropriate to a greater extent. The current data agree with the recent studies that found the ANN model was superior to RSM, recording lower values of RMSE, MAD, and SSE and greater values of R^2^^21,69^. Consequently, ANN has a better generalization capability than CCFCD.

### Validation of CCFCD, and ANN models

The experimental validation of CCFCD and ANN models for the maximization of CNPs biosynthesis by *Streptomyces microflavus* strain NEAE-83 was checked using the computed theoretical values of the four tested factors. The desirability function was used to find the best conditions for maximum CNPs biosynthesis. To validate both models, the optimum theoretical values that maximize the CNPs biosynthesis were estimated by both models to be 5.5 initial pH, 1.3% chitosan, 40 °C, and 12 h incubation period. The corresponding estimated CNPs biosynthesis based on CCFCD, and ANN were 9.29, and 9.44 mg/mL, respectively. Under these conditions, the theoretical values were assessed experimentally. The experimental value of the microbially synthesized CNPs by *Streptomyces microflavus* strain NEAE-83 was 9.52 mg/mL, confirming a great precision degree of both models. However, the estimated value by ANN was much closer to the experimental one than that estimated by CCFCD, proving again that ANN has an advanced predictive power than the CCFCD model. This supposition, in fact, provides a robust indication for the design's and modeling procedure's fitness, applying the tested arrays of the four factors in the bacterial CNPs biosynthesis process.

The current optimum pH of 5 was previously reported as its superior over that at neutral range at pH 7, nanoparticles at pH lesser than 7 tend to be smaller and globular, whereas the particles created at a pH outside 7 tend to be non-spherical and bigger in size or agglomeration of small particles^70^. Our characterization studies support such results.

Regarding the current chitosan concentration of 1.295 mg/mL, chitosan intensity powerfully impacts the size and growth of the nanoparticles. Chitosan concentration at 1.0 mg/mL was among the best conditions to generate CNPs with the lowest size (95 nm) compared to the higher concentrations^71^.

The reaction temperature at 40 °C in the current study could be considered suitable from a biotechnological point of view. Since the reaction temperature plays a key role in particle development, and shape/size control, where, the reaction temperature can greatly influence the reaction rate, and hence the particle features^72^. Current characterization findings support the data recovered from the optimization process regarding shape and size.

The current incubation period of 12 h is a reasonably suitable time since, Saifful and Shahidan^73^ reported that the prolongation of incubation time (18 h) generated a higher average size of nanoparticles compared with the shorter time (2 h). The same conclusion was reported also by Vaezifar et al*.*^71^.

In contrast to the single-step calculation models, the high precision of ANN predictive capability may be owing to its wide ability to estimate the nonlinearity of the system^20,21^. However, there are some drawbacks of ANN modeling, of which ANN devours a comprehensive computational period through iterations of estimates, and the organized nature cannot demonstrate the contributions and the significance of each factor in the model, thus factors in the model cannot be reduced or eliminated from the model^21^.

### Antimicrobial activity assay of chitosan and the prepared CNPs

**Figure 14:**
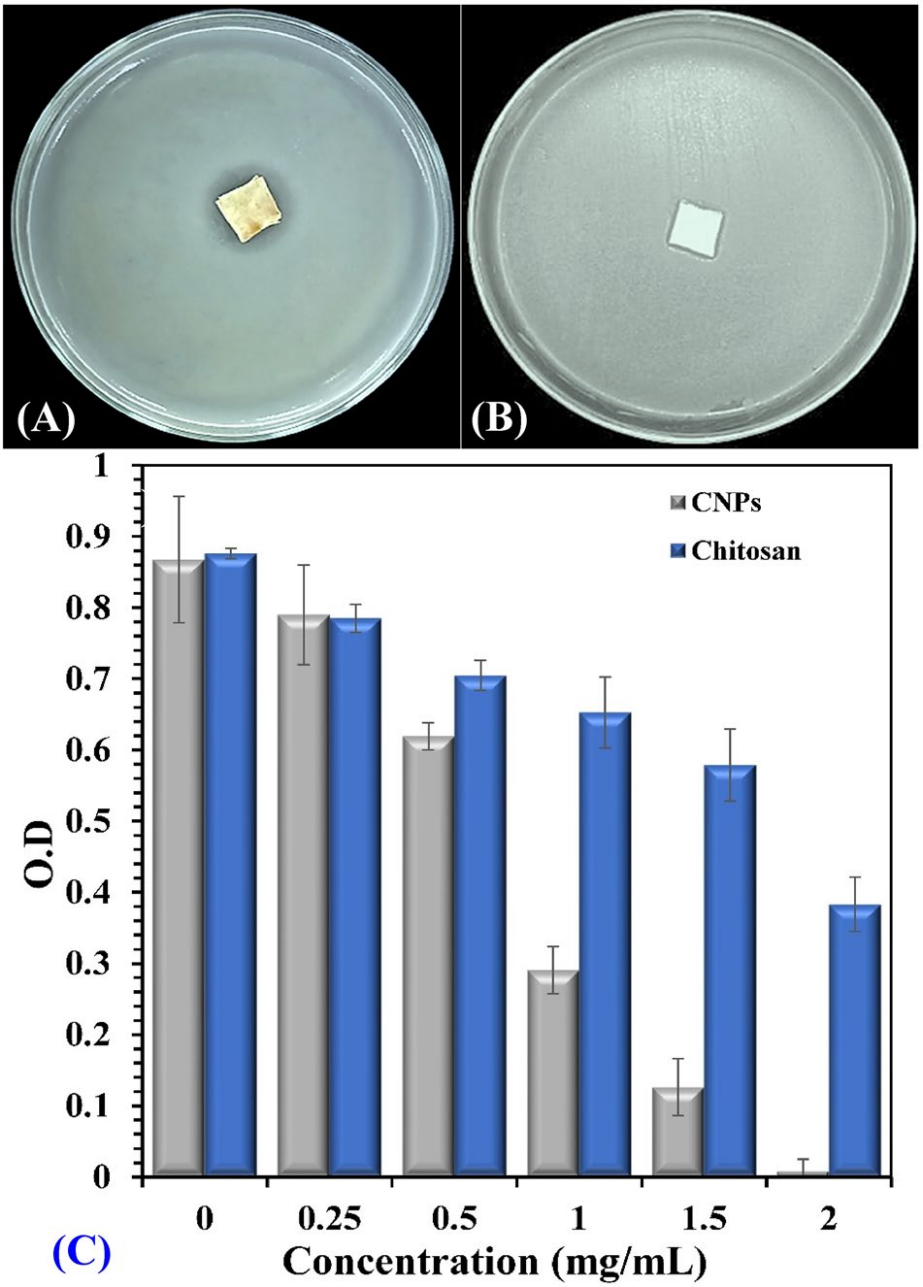
Antimicrobial activity assay of (**A**) CNPs and, (**B**) chitosan standard against *Pectobacterium carotovorum*, (**C**) The minimum inhibitory concentration (MIC) of chitosan and CNPs biosynthesized by *Streptomyces microflavus* strain NEAE-83.

The antibacterial activity of chitosan and the prepared CNPs was investigated against the plant pathogenic bacteria *Pectobacterium carotovorum* using agar diffusion and growth inhibition assay. CNPs showed a significant inhibition zone (19 mm) against the pathogenic bacteria when compared with chitosan solution (11 mm), as shown in Fig. 14A,B. CNPs markedly suppressed the bacterial growth upon increasing its concentration in a liquid medium. The lowest concentration of CNPs which can prevent the growth of bacteria was recorded at 2 mg/mL.

Chitosan is well-known as a natural, non-toxic polymer, that has antibacterial activity. In the agriculture filed, chitosan can be used as a natural pesticide for preventing and controlling plant diseases^74,75^. Chitosan in nanoscale provided a novel and unique activities including antimicrobial, antitumor effects, and drug delivery system^71^. In the current study, the CNPs showed higher antibacterial activity than chitosan against *Pectobacterium carotovorum*. This is due to the uniform spherical shape and smaller size of CNPs in comparison to chitosan. The polycationic character of CNPs enables them to interact with the negatively charged bacterial cell wall greater than chitosan itself. Besides, the CNPs have large surface area which facilitates it to be tightly absorbed onto the bacterial surface. This causes disruption to the cell membrane, and leakage of intercellular compounds which in turn affects cell viability^51^. Penetration of the CNPs into the bacterial cell causes DNA damage and prevents protein synthesis from RNA^76^. In agreement with other investigations, our results showed that CNPs inhibit the growth of *Pectobacterium carotovorum* with a diameter of 19 mm. Ahmed et al.^77^ reported that the CNPs inhibit the growth of four isolates of gram- positive (*Staphylococcus aureus, Bacillus subtilis*) and gram-negative bacteria (*Escherichia coli*, *Pseudomonas aeruginosa*) with a diameter range from 9 to 16 mm. In another study by Divya et al.^78^, they reported that the diameter of the inhibition zone produced by CNPs against *klebsiella pneumoniae*, *Staphylococcus aureus*, *Pseudomonas aeruginosa* and *Escherichia coli* ranged between 12 and 16 mm.

The antimicrobial activity of CNPs various depending on a number of factors like type, molecular weight, degree of polymerization of chitosan, the solvent used, temperature and pH value. CNPs also exhibit dose-dependent and species-specific antimicrobial properties^79^. The gram-positive bacteria showed more sensitivity against CNPs than gram-negative bacteria. Where, the cell wall of gram-positive bacteria is composed of a thick layer of lipoprotein, and phospholipids, that lead to increasing the negative charge on the cell surface^80^. In this study, the minimum inhibition concentration of CNPs against *Pectobacterium carotovorum* was 2.0 mg/mL (Fig. 14C). Aliasghari et al.^81^ reported that the minimum inhibitory concentration (MIC) of CNPs for four strains of *Streptococcus* was ranged from 1.25 to 2.5 mg/mL. Similarly, CNPs have a bactericidal effect against seven bacterial strains at concentrations various from 0.125 to 5 mg/mL^77^.

## Conclusions

A pioneer microbial-based plan for the synthesis of CNPs utilizing a novel *S. microflavus* was reported. The resulting CNPs were fully identified using electron microscopy investigation, EDXS, mapping FTIR spectroscopy, thermogravimetry, and DSC, all confirming the high quality of the product. The process parameters were studied and optimized to maximize the yield of CNPs using CCFCD and artificial intelligence for the first time in this kind of biosynthesis. Nevertheless, the challenges to be resolved and elucidated are the economics of the large-scale production process, and their cytotoxicity in comparison to the ordinary CNPs, before being permitted as a profitable outcome.

## Materials and methods

### Actinomycete and cultivation conditions

The usual dilution plate technique was used to isolate *Streptomyces* sp. strain NEAE-83, from a soil sample gathered from El Warraq, Giza Governorate, Egypt, on Petri dishes containing starch nitrate agar medium of the next composition (g/L): starch, 20; MgSO_4_.7H_2_O, 0.5; KNO_3_, 2; NaCl, 0.5; K_2_HPO_4_, 1; FeSO_4_.7H_2_O, 0.01; CaCO_3_, 3; agar, 20. Petri dishes were incubated at 30 °C for 7 days. The isolated strains were maintained in 20% (v/v) glycerol as spore suspensions at -20 °C.

### Preparation of chitosan solution

Low molecular weight mass (Sigma-Aldrich) was liquified at 2% (w/v) with 0.5% (v/v) acetic acid and then raised to pH 4.8–5.0 with 1 N NaOH under magnetic stirring for 24 h and completed up to a 200 mL.

### Biological preparation and detection of CNPs

The optimal growth medium of the actinomycete *Streptomyces* sp. strain NEAE-83 (inoculum concentration) were K_2_HPO_4_ (0.05%), MgSO_4_ (0.05%), NaCl (0.05%), KNO_3_ (0.1%), FeSO_4_ 0.7H_2_0 (0.001%), yeast extract (0.03%), starch (2%), and pH 7.5. A volume of 2 mL of culture filtrate was blended with 1 mL of chitosan solution (1%) and incubated at 30 °C for 12–36 h under shaking at 150 rpm to obtain an opalescent solution.

The colloidal suspension was centrifuged (10 min at 10,000×*g*). The resulting precipitate was washed two-times to eliminate the unreacted ingredient. The resultant was dissolved in 1% acetic acid again. The bio-synthesized CNPs were determined and monitored through UV–visible spectrum by detecting the peak absorbance by the double beam Optizen Pop-UV/VIS spectrophotometer at an array between 200 and 400 nm. The precipitate was then freeze-dried. A calibration curve was made from CNPs, using known concentrations (mg/mL) through dissolving in 1% acetic acid to estimate the net yield of CNPs.

### Identification of *Streptomyces* sp. strain NEAE-83

#### Cultural features and cell morphology

The spore surface and spore chain patterning of the bacterium were investigated on starch nitrate agar after growing at 30 °C for 14 days. The dehydrated gold-coated bacterium was examined at various amplifications with analytical SEM (Jeol JSM-6360 LA).

#### Physiological and antimicrobial characteristics

The physiological features e.g., production of diffusible and melanoid pigments (on broth medium of yeast extract tryptone, and agar media of both peptone-yeast extract-iron, and tyrosine), coagulation and peptonization of milk, starch hydrolysis, chitosanase, L-asparaginase, protease, uricase, cellulase, nitrate reduction, gelatin liquefaction, H_2_S production, and NaCl tolerance, were determined^2^. The ability of *Streptomyces* sp. strain NEAE-83 to hinder the growth of some bacteria (*Staphylococcus aureus*, *Pseudomonas aeruginosa*, *Klebsiella* sp., and *Escherichia coli*), and fungi (*Fusarium solani*, *F. oxysporum*, *Alternaria solani*, *Bipolaris oryzae*, and *Rhizoctonia solani*) was also clarified^2^.

### Molecular identification

The cells were harvested into a 2 mL microcentrifuge tube by centrifugation at 5000 × g for 10 min, the supernatant was removed. The genomic DNA was prepared as described by the method of PCR amplification of the 16S rRNA gene was performed using the protocol of El-Naggar et al.^17^. The prepared lysate was purified using a GeneJET^TM^ Genomic DNA Purification Column. The PCR amplification reaction was performed in a mixture of 25 μl Maxima Hot Start PCR Master Mix (2X), 1 μl of 20 pmol 16S rRNA forward primer 27f. (5′-AGAGTTTGATCMTGCCTCAG-3′), 1 μl of 20 pmol 16S rRNA reverse primer 1492 r (5′-TACGGYTACCTTGTTACGACTT-3′), 5 μl template DNA, and 18 μl water, nuclease-free.

The thermal cycling of the PCR-apparatus was programmed as recommended (10 min at 95 °C for initial denaturation and enzyme activation, followed by 35 amplification cycles of 30 s at 95 °C, 1 min of annealing at 65 °C, and 1.5 min at 72 °C, followed by final extension for 10 min at 72 °C). After that, the mixture of PCR product was run through the agarose gel electrophoresis and purified by GeneJET™ PCR Purification Kit (Thermo K0701). The resulting purified PCR was sequenced at GATC Company by using ABI 3730xl DNA sequencer using both forward and reverse primers. The 16S rRNA gene sequence of *Streptomyces* sp. strain NEAE-83 (1214 bp) was dropped in the GenBank database to retrieve the accession number.

The gene sequence of *Streptomyces* sp. strain NEAE-83 was compared to the sequences of those other *Streptomyces* strains on the GenBank (EMBL, DDBJ, and PDB) databases, and the 16S rRNA gene sequences was used for constructing a phylogenetic tree (neighbor-joining tree) to display the relationships between *Streptomyces* sp. strain NEAE-83 and interrelated *Streptomyces* species. MEGA-X software package was used.

### Description of CNPs

#### Electron microscopy investigation

The generated CNPs was coated by gold sputter (SPI-Module), then the morphology, size, and construction were inspected by SEM (model JEOL-JSM-IT200) at 20 kV. Another morphological examination of CNPs was performed by TEM. Samples of the generated CNPs were inspected with the TEM unit (TEM; JEM-2100 Plus, JEOL Ltd., Japan).

#### Analyses of EDXS

The characterization and elemental configuration of CNPs were discovered by EDXS. To acquire in-depth knowledge regarding the CNPs, the electron beam of the SEM was used to examine individual CNPs. The obtained data using the program were analyzed.

#### Mapping analysis

Mapping analysis was performed with the aid of SEM to take an overview image of CNPs for studying their composition and distribution.

#### Particle size analysis

Dynamic laser scattering (DLS) using the N5 submicron Beckman Coulter particle size analyzer was used to measure the particle size distribution of the CNPs.

#### Zeta potential

The surface charge properties of the CNPs were quantified using ζ-potential through the software of Malvern analytical Zetasizer (version 7.13) fortified with a laser doppler and distinguished by the phase analysis light scattering. The determinations were carried out at 25 °C, with CNPs taken in their liquid-state^82^.

#### FTIR spectroscopy

The surface properties of the CNPs were conducted through FTIR spectroscopy, using Shimadzu FTIR-8400 S spectrophotometer. CNPs were crushed using KBr pellets, then the FTIR spectrum was conducted at a resolution of 1 cm^−1^ through the range between 4500 and 500 cm^−1^.

#### Pattern of XRD

The structural properties of CNPs were elucidated using XRD, which is a vital practice for discovering the XRD pattern. The diffractometer (Bruker D2 Phaser 2nd Gen) was used, by which, the X-ray works with a Cu anode having 30 kV and 10 mA. The intensity of diffraction was determined at 25.7 °C with 2°/min of scanning rate for 2θ = 10–50 of Khan et al.^83^.

#### CNPs thermal behavior

The CNPs sample was prepared by drying at 60 °C for 1 h before mounting in a platinum sample pan. TGA was accomplished with a 20 mL min^−1^ flow rate in the range from 25 to 500 °C. The thermo-analyzer (50-H) was used under a nitrogen atmosphere at an increase rate of 10 °C min^−1^. The diagram was plotted as the percentage of weight loss vis temperature.

The CNPs pyrolysis pattern of DSC was estimated after drying for 1 h at 60 °C. CNPs sample was mounted in the aluminum pan. The DSC (60-A) test was accomplished in nitrogen atmosphere settings with flow and heating ratios of 30 mL min^−1^ and 10 °C min^−1^, respectively. The thermogram behavior was explored between 25 to 500 °C. The DSC upper temperature was the original breakdown temperature of CNPs, as reported by the thermogravimetric examination. The diagram was plotted as temperature vis heat flow.

### Matrix of central composite face-centered design (CCFCD)

Central composite face-centered design (CCFCD) is an effective approach that is extensively used in optimization procedures because it gives enough information for confirming the model's accuracy without needing a huge number of experiments, minimizing the overall cost of the experiment^84^. The matrix of CCFCD was created to determine the greatest combination of the tested factors that affect CNPs biofabrication by *Streptomyces microflavus* strain NEAE-83. Three levels of every variable (coded as − 1, 0, + 1) were applied. The design was assigned with 5 center points. Accordingly, thirty runs were generated (Table 2). The coded values were used since coding is better to identify the relative impact of the factors by comparing the factor coefficients. The coded values of the investigated factors were estimated from the actual value by the next function:

1 $${x}_{i}=\left({X}_{i}- {X}_{0}\right)/{\Delta X}_{i}$$

where *x*_*i*_ is the coded value of a tested factor, *Xi* is the real value of a tested factor, X_0_ is the real value of the tested factor at the middle point and *∆X*_*i*_ is the step-change in the actual value of the variable *i*.

The actual levels were initial pH; 4.5–5.5, initial chitosan concentration; 1–2%, temperature; 30–40 °C, and incubation period; 12–36 h. The linear, quadratic, and mutual effects of the designated process factors were estimated to discover the connection between CNPs generation and the optimum level of the examined factors that influence the biosynthesis process. The following second-order polynomial equation was used:

2 $$Y={\beta }_{0}+\sum_{i}{\beta }_{i}{X}_{i}+\sum_{ii}{\beta }_{ii}{{X}_{i}}^{2}+\sum_{ij}{\beta }_{ij}{X}_{i}{X}_{j}$$

where *Y* is the CNPs biosynthesis using *Streptomyces microflavus* strain NEAE-83, *X*_*i*_ is the process variable level, *β*_*0*_ is the coefficients of regression, *β*_*i*_ is the linear coefficient, *β*_*ij*_ is the mutual coefficients and *β*_*ii*_ is the quadratic coefficients.

### ANN modeling of CNPs biosynthesis

A completely linked neural network program was built, employing various nodes within their layers. All nodes have an NTanH activation function. For developing a prediction model using artificial intelligence, the CCFCD matrix, and experimental data (Table 2), were employed to train the connected multilayer perceptron of ANN. The CCFCD data were divided into training (to create neural weights and minimize prediction error), validation (to choose the best model and stop training), and testing (for assessment of ANN forecast competence).

The design ANN topology is composed of the input layer fixed by the four independent factors and the output layer that has only one fixed neuron (CNPs biosynthesis by *Streptomyces microflavus* strain NEAE-83). In-between, there was the hidden layer(s) that was examined using several criteria, including the number of neurons, holdback propagation ratio, and learning rates. The ANN topology is designated as 4-h-1.

The trial-and-error procedure was used for model selection, and evaluation of machine learning efficacy, which was determined based on RMSE, MAD, SSE, and R^2^ tests. Another, the predicted outputs were very near to the actual response of CNPs biosynthesis. The fitness of CCFCD and ANN models was compared with the corresponding experimental values.

### Software and statistical analysis

The software, Design Expert (version 13, Stat-Ease, Minneapolis, USA), was utilized to set up the CCFCD design matrix and perform the statistical examinations. The machine learning procedure, construction of the ANN topology, and statistical examinations were performed with aid of the software package; JMP pro, version 16.2 (JMP, SAS Institute Inc., Cary, NC), through which the training, validating, and testing processes were performed.

### Antimicrobial activity assay for chitosan standard and CNPs using agar diffusion method

Stock-culture of *Pectobacterium carotovorum* (kindly provided by Dr. Muhammad Zayed, Botany and Microbiology Department, Menoufia University, Egypt) was sub-inoculated into sterile Luria–Bertani (LB) broth medium and incubated overnight at 30 °C with shaking. After 24 h, the bacterial suspension was adjusted to 10^8^ CFU/mL (0.5 McFarland Standard) with sterile saline solution. One hundred microliters of the bacterial suspension were spread on the surface of LB agar plate using sterile swabs. Then, a 1 cm-diameter piece of fabric saturated with 20 µg CNPs and another piece of fabric saturated with 20 µg of chitosan standard were placed on the surface of the inoculated plates. The plates were kept under refrigeration to improve material diffusion into agar, and the plates were incubated at 30 °C for 18 h.

### Broth microdilution assay for chitosan standard and CNPs

The MIC of CNPs was determined by the microdilution method against *Pectobacterium carotovorum*. The stock solutions of chitosan standard and CNPs were serially diluted from 0.25 to 2.0 mg/mL in LB broth medium and dispensed uniformly into a sterile 96-well microtiter plate in triplicate for each concentration. Each well was inoculated with 20 µL of a bacterial suspension (10^5^ CFU/well). Wells containing culture medium and different concentrations of chitosan standard and CNPs without inoculation were used as negative controls. The bacterial growth was estimated by turbidity measurements at 550 nm after overnight incubation.

## Supplementary Information

Supplementary Information.

## Data Availability

All data generated or analyzed during this study are included in this published article and its supplementary information file.
