# Supplementary material for: Innovative biosynthesis, artificial intelligence-based optimization, and characterization of chitosan nanoparticles by Streptomyces microflavus and their inhibitory potential against Pectobacterium carotovorum
Source: Sci Rep. 2022 Dec 17;12:21851. doi: 10.1038/s41598-022-25726-w (PMC9759534; doi:10.1038/s41598-022-25726-w)
Supplement: Supplementary file 1 — Supplementary Information. [file 41598_2022_25726_MOESM1_ESM.pdf]

Original Article

Innovative biosynthesis, artificial intelligence-based optimization, and characterization of chitosan nanoparticles by *Streptomyces microflavus* and their inhibitory potential against *Pectobacterium carotovorum*

Noura El-Ahmady El-Naggar[1], Shimaa I. Bashir [2], Nashwa H. Rabei[1], WesamEldin I.A. Saber[3]

[1] Department of Bioprocess Development, Genetic Engineering and Biotechnology Research Institute, City of Scientific Research and Technological Applications (SRTA-City), New Borg El-Arab City 21934, Alexandria, Egypt.

[2]Department of Plant Protection and Biomolecular Diagnosis, Arid Land Cultivation Research Institute, City of Scientific Research and Technological Applications (SRTA-City), New Borg El-Arab City 21934, Alexandria, Egypt.

[3]Microbial Activity Unit, Department of Microbiology, Soils, Water and Environment Research Institute, Agricultural Research Center, 12619, Giza, Egypt.

Correspondence

Dr. Noura El-Ahmady Ali El-Naggar

E-mail: nelahmady@srtacity.sci.eg

nouraalahmady@yahoo.com

Tel: (002)01003738444 Fax: (002)03 4593423

Complete gel for Figure 4 A

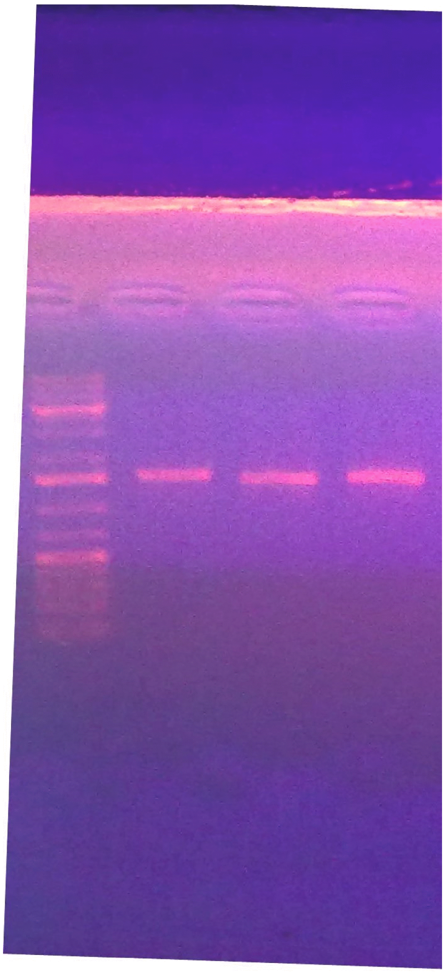

Uncropped image of agarose gel of PCR product bands of the amplified 16S fragment.
